# Assessing the effects of multiple infections and long latency in the dynamics of tuberculosis

**DOI:** 10.1186/1742-4682-7-41

**Published:** 2010-11-08

**Authors:** Hyun M Yang, Silvia M Raimundo

**Affiliations:** 1 UNICAMP-IMECC. Departamento de Matemática Aplicada, Praça Sérgio Buarque de Holanda, 651, CEP: 13083-859, Campinas, SP, Brazil; 2 USP-FM. Disciplina de Informática Médica, Instituto Oscar Freire, Rua Teodoro Sampaio, 115, CEP: 05405-000, São Paulo, SP, Brazil

## Abstract

In order to achieve a better understanding of multiple infections and long latency in the dynamics of *Mycobacterium tuberculosis *infection, we analyze a simple model. Since backward bifurcation is well documented in the literature with respect to the model we are considering, our aim is to illustrate this behavior in terms of the range of variations of the model's parameters. We show that backward bifurcation disappears (and forward bifurcation occurs) if: (a) the latent period is shortened below a critical value; and (b) the rates of super-infection and re-infection are decreased. This result shows that among immunosuppressed individuals, super-infection and/or changes in the latent period could act to facilitate the onset of tuberculosis. When we decrease the incubation period below the critical value, we obtain the curve of the incidence of tuberculosis following forward bifurcation; however, this curve envelops that obtained from the backward bifurcation diagram.

## Background

Infectious diseases in humans can be transmitted from an infectious individual to a susceptible individual directly (as in childhood infectious diseases and many bacterial infections such as tuberculosis) or by sexual contact as in the case of HIV (human immunodeficiency virus). They can also be transmitted indirectly by vectors (as in dengue) and intermediate hosts (as in schistosomiasis). According to the natural history of diseases, an incubation period followed by an infectious period has to be considered a common characteristic. Numerous viral infections confer long-lasting immunity after their infectious periods, mainly because of immunological memory [1]. However, in many bacterial infections, antigenically more complex than viruses, the acquisition of acquired immunity following infection is neither so complete nor confers long-lasting immunity. Hence, in most viral infections, a single infection is sufficient to stimulate the immune system and elicit a lifelong response, while multiple infections can occur in diseases caused by bacteria.

The simplest quantitative description of the transmission of infections is the mass action law; that is, the likelihood of an infectious event (infection) is proportional to the densities of susceptible and infectious individuals. Essentially, this law oversimplifies the acquisition of infection by susceptibles from micro-organisms excreted by infectious individuals into the environment (aerial transmission), or present in the epithelia (infection by physical contact) or the blood (transmission by sexual contact or transfusion) of infectious individuals.

In this paper we deal with the transmission dynamics of tuberculosis. Tuberculosis (TB) is caused by *Mycobacterium tuberculosis *(MTB), which is transmitted by respiratory contact. This presents two routes for the progression to disease: primary progression (the disease develops soon after infection) or endogenous reactivation (the disease can develop many years after infection). After primary infection, progressive TB may develop either as a continuation of primary infection (fast TB) or as endogenous reactivation (slow TB) of a latent focus. In some patients, however, disease may also result from exogenous reinfection by a second strain of MTB. There are reports of exogenous reinfection in the literature in both immunosuppressed and immunocompetent individuals [2]. Martcheva and Thieme [3] called the exogenous reinfection 'super-infection'.

To what extent simultaneous infections or reinfections with MTB are responsible for primary, reactivation or relapse TB has been the subject of controversy. However, cases of reinfection by a second MTB strain and occasional infection with more than one strain have been documented. Shamputa *et al. *[4] and Braden *et al. *[5] investigated that in areas where the incidence of TB is high and exposures to multiple strains may occur. Although the degree of immunity to a second MTB infection is not known, simultaneous infection by multiple strains or reinfection by a second MTB strain may be responsible for a portion of TB cases.

A very special feature of TB is that the natural history of the disease encompasses a long and variable period of incubation. This is why a super-infection can occur during this period, overcoming the immune response and resulting in the onset of disease. When mathematical modelling encompasses the natural history of disease (the onset of disease after a long period since the first infection) together with multiple infections during the incubation period to promote a 'short-cut' to disease onset, a so-called 'backward' bifurcation appears (see Castillo-Chavez and Song [6] for a review of the literature associated with TB models). Another possible 'fast' route is due to acquired immunodeficiency syndrome (AIDS) [7-9].

Our aim is to understand the interplay between multiple infections and long latency in the overall transmission of TB. Another goal is to assess how they act on immunosuppressed individuals. Since the backward bifurcation is well documented in the literature, we focus on the contributions of the model's parameters to the appearance of this kind of bifurcation.

This paper is structured as follows. In the following section we present a model that describes the dynamics of the TB infection, which is analyzed in the steady state with respect to the trivial and non-trivial equilibrium points (Appendix B). In the third section we assess the effects of super-infection and latent period in TB transmission. This is followed by a discussion and our conclusions.

## Model for TB transmission

Here we present a mathematical model of MTB transmission. In Appendix A, we briefly present some aspects of the biology of TB that substantiate the hypotheses assumed in the formulation of our model.

There are many similarities between the ways by which different infectious diseases progress over time. Taking into account the natural history of infectious disease, in general the entire population is divided into four classes called susceptible, latent (exposed), infectious and recovered (or immune), whose numbers are denoted, respectively, by *S*, *E*, *Y *and *Z*.

With respect to the acquisition of MTB infection, we assume the true mass action law, that is, the per-capita incidence rate (or force of infection) *η *is defined by *η = βY/N*, where *β *is the transmission coefficient and *N *is the population size. Hence the development of active disease varies with the intensity and duration of exposure. Susceptible (or naive) individuals acquire infection through contact with infectious individuals (or ill persons in the case of TB) releasing infectious particles, where the incidence is *ηS*. After some weeks, the immune response against MTB contains the mycobacterial infection, but does not completely eradicate it in most cases. Individuals in this phase are called exposed, that is, MTB-positive persons.

The transmission coefficient *β *depends among a multitude of factors on the contacts with infectious particles and duration of contact. Let us consider this kind of dependency as

β=kωχϱ,

where *k *is the constant of proportionality, ω is the frequency of contact with infectious particle, χ is the duration of contact and ϱ is the amount of inhaled MTB. It is accepted that persons with latent TB infection have partial immunity against exogenous reinfection [10]. This means that super-infection can occur among exposed individuals, but to be successful the inoculation must involve more mycobacteria than the primary infection. We assume that multiple exposure can precipitate progression to disease, according to a speculation [11]. Let us, for simplicity, assume that the minimum amount of inoculation needed to overcome the partial immune response is given by a factor *P*, with *P *> 1 (*P *= 1 means absence of immune response, while if *P *< 1, primary infection facilitates super-infection, that is, increases the risk of active disease and acts as a kind of anti-immunity). In terms of parameters we have *ϱ*^*e*^*=Pϱ*, and we assume that all other factors (ω and χ) are unchanged. This assumption gives the super-infection incidence rate as *p*η, where *p *= 1/*P *(hence 0 <*p *< 1, if we exclude anti-immunity) is a parameter measuring the degree of partial protection, and η is the per-capita incidence rate in a primary infection. The lower the value of *p*, the greater the immune response mounted by exposed persons, which is the reason why much more inoculation is required in a posterior infection to change their status (*P *is high).

Susceptible individuals as well as latently infected persons can progress to disease in a primary infection. If the level of inoculation is lower, the immune response is quite efficient and primary infection ensues in the latently infected person. However, if the inoculation is increased, say above a factor *P' (p' = 1/P'*), this amount can overcome the immune response and lead to primary TB. In terms of parameters we have *ϱ*^*s*^*=P'ϱ*, and we assume again that that all other factors (ω and χ) are unchanged. Naturally we have *p' < 1*, because naive susceptible individuals are inoculated with ϱ amount of MTB to be latently infected. It is true that susceptible individuals are likely to be at greater risk of progressing to active TB than latently infected individuals; hence, to be biologically realistic, we must have *p < p'*.

According to the natural progression of the disease, after a period of time γ^*-1*^, where γ is the incubation rate, exposed individuals manifest symptoms. Among these individuals, we assume that super-infection results in a 'short cut' to the onset of disease owing to a huge number of inoculated bacteria, instead of completing the full period of time γ^*-1*^. Individuals with TB remain in the infectious class during a period of time δ^*-1*^, where δ is the recovery rate. In the case of TB, the recovery rate can be considered to include antituberculous chemotherapy, which results in a bacteriological cure. The presence of memory T cells protects treated individuals for extended periods. Finally, let us assume that recovered (or MTB-negative) individuals can be reinfected according to the incidence rate *qη*, where the parameter *q*, with 0≤*q*≤1, represents a partial protection conferred by the immune response. The interpretation of *q *is quite similar to the parameter *p*. Note that *q *= 0 mimics a perfect immune system (immunological memory is everlasting) that avoids reinfection (we have a susceptible-exposed-infectious-recovered type of model), while *q *= 1 (immunological memory wanes completely) describes the case where the immune system confers no protection (we have a susceptible-exposed-infectious-susceptible type of model), in which case we can define a new compartment *W *that comprises the *S *and *Z *classes of individuals (*W *= *S*+*Z*). For intermediate values, 0 <*q *< 1, the model considers a lifelong and partial immune response, because we do not allow the return of individuals in the recovered class to the susceptible class, but they can be re-infected. The case *q *> 1 represents individuals who have previously had TB disease are may be at high risk of re-infection leading to future disease episodes [11].

Cured (MTB-negative) individuals are also at risk of progressing to active TB in an infective event with a higher level of inoculation. As we argued for susceptible and latently infected individuals, this event is described by the parameter *q'*. Because relapse to TB requires more inoculation in cured persons than infection in latently infected persons, we must have *q' < q*.

On the basis of the above assumptions, we can describe the propagation of MTB infection in a community according to the following system of ordinary differential equations

$$\left\{ \begin{array}{l} \frac{dS}{dt}=\phi -\left(1+p'\right)\;\eta S-\mu S\\ \frac{dE}{dt}=\eta S+q\eta Z-p\eta Z-\left(\mu +\gamma \right)\;E\\ \frac{dY}{dt}=p'\eta S+q'\eta Z+p\eta E-\left(\mu +\delta +\alpha \right)\;Y\\ \frac{dZ}{dt}=\delta Y-\left(q+q'\right)\;\eta Z-\mu Z, \end{array}\right.$$

where all the parameters are positively defined, and the terms *p'ηS *and *q'ηZ *are, respectively, primary progress to TB in susceptible persons, and direct relapse into infection in individuals cured of TB. The parameters μ and α are the natural and additional constant mortality rates and *ϕ *is the overall input rate, which describes changes in the population due to birth and net migration. To maintain a constant population, we assume that the overall input rate *ϕ *balances the total mortality rate, that is, *ϕ = μN+αY*, where *N *is now the constant population size, *N *= *S*+*E*+*Y*+*Z*. In the literature, primary TB is considered a proportion of total incidence, that is, *(1-l)ηS*, where *l *is a proportion, instead of (*1 + p')ηS *(see, for instance, [6,12]).

Using the fact that *N *is constant, we introduce the fractions (number in each compartment divided by *N*) of susceptible, exposed, infectious and recovered individuals as *s*, *e*, *y *and *z*, respectively. Hence the system of equations can be rewritten:

(1) $$\left\{ \begin{array}{l} \frac{ds}{dt}=\mu +\alpha y-\left(1+p'\right)\beta ys-\mu s\\ \frac{de}{dt}=\beta ys+q\beta yz-p\beta ye-\left(\mu +\gamma \right)\;e\\ \frac{dy}{dt}=p'\beta ys+q'\beta yz+p\beta ye+\gamma e-\left(\mu +\delta +\alpha \right)\;y\\ \frac{dz}{dt}=\delta y-\left(q+q'\right)\beta yz-\mu z, \end{array}\right.$$

where *s+e+y+z *= 1. This system of equations describes the propagation of infectious disease in a community with constant population size, that is, $\frac{dN}{dt}=0$. The set of initial conditions *G *supplied to this dynamical system is

$$G=\left(s_{0}\;,\;e_{0}\;,\;y_{0}\;,\;z_{0}\;\right)\;.$$

Notice that the equation related to the recovered individuals can be decoupled from the system by the relationship *z *= 1-*s*-*e*-*y*.

The system of equations (1) is not easy to analyze because of several non-linearities. Instead, we deal with a simplified version of the model, disregarding primary progression to TB and relapse to TB among cured individuals. The system of equations we are dealing with here is

(2) $$\left\{ \begin{array}{l} \frac{ds}{dt}=\mu +\alpha y-\beta ys-\mu s\\ \frac{de}{dt}=\beta ys+q\beta yz-p\beta ye-\left(\mu +\gamma \right)\;e\\ \frac{dy}{dt}=p\beta ys+\gamma e-\left(\mu +\delta +\alpha \right)\;y\\ \frac{dz}{dt}=\delta y-q\beta yz-\mu z. \end{array}\right.$$

In the Discussion we present the reasoning behind these simplifications. Our aim is to assess the effects of super-infection and re-infection in a MTB infection that presents long period of latency.

The analytical results of system (2) are restricted to an everlasting and perfect immune response (*q *= 0, since the immune system mounts cell-mediated response against MTB, leaving an immunological memory after clearance of invading bacteria), and to a quickly waning immune response (*q *= 1, absence of immune response). For other values of *q*, numerical simulations are performed. As pointed out above, when *q *= 1, we can define a new compartment *w*, where *w *= *s*+*z*, combining persons who are susceptible (*s*) with those who are MTB negative but do not retain immunity (*z*), to yield a reduced system given by

(3) $$\left\{ \begin{array}{l} \frac{dw}{dt}=\mu +\left(\delta +\alpha \right)\;y-\beta yw-\mu w\\ \frac{de}{dt}=\beta yw-p\beta ye-\left(\mu +\gamma \right)\;e\\ \frac{dy}{dt}=p\beta ye+\gamma e-\left(\mu +\delta +\alpha \right)\;y\;. \end{array}\right.$$

The system of equations (3) describes super-infection (*p*) precipitating the onset of disease after a long period of latency (γ), and reinfections (*q*) among MTB-negative individuals whose immunological memory wanes. This system was used by [13], with α = 0, to describe TB transmission taking into account the 'fast' and 'slow' evolution to the disease after first infection with MTB: the parameter γ represents the 'slow' onset of disease, while super-infection (parameter *p*) is used as a descriptor of 'fast' progression to TB. Immunosuppressed individuals may have increased γ, and this is another fast progression to TB.

Our intention is to assess the effects of varying the model's parameters in the backward bifurcation. We analyze the system (2) in steady states.

## Assessing the effects of multiple infections and latent period on MTB infection

The analysis of the model is given in Appendix B, where all equations referred to in this section are found. On the basis of those results, we assess the role played by super-infection (described by *p*), reinfection (*q*) and long latent period (γ^*-1*^) in the dynamics of MTB infection. We discuss some features of the model and numerical results are also presented.

First, we analyze *p*~0, absence of super-infection. The results from this approach will be compared with the next two cases. Secondly, we assess the case γ~0, that is, the onset of TB occurs after a period longer than the human life-span. This case deals with human hosts developing a well-working immune response. Finally we return to the case γ > 0 and *p *> 0 in order to elicit TB transmission.

### Modeling TB without super-infection

Here super-infection is not considered by letting *p *= 0 (this is the limiting case *P→∞*, or *p→0*) in the system of equations (2). One of the main features of microparasite infections [14] is that exposed individuals enter the infectious class after a period of time, and super-infection does not matter during this period. Mathematical results are readily available (see for instance [15]) so we reproduce them briefly here.

This case (*p *= 0 and γ > 0) has, in the steady state, the trivial equilibrium point *P^0 ^*= (1,0,0,0) which is stable when *R*_0 _< 1, otherwise unstable, as shown in Appendix B.

With respect to the non-trivial equilibrium point, we present two special cases: *q = 0 *and *q *= 1.

When *q *= 1, a unique positive root exists for the polynomial $Q\left(\beta \bar{y}\right)$, given by equation (B.7), where the coefficients, given by equation (B.8) are, letting *p *= 0,

$$\left\{ \begin{array}{l} a_{2}=0\\ a_{1}=\mu +\gamma +\delta +\alpha \\ a_{0}=\left(\mu +\gamma \right)\;\left(\mu +\delta +\alpha \right)\;\left(1-\frac{\beta }{\beta_{0}}\right)\;, \end{array}\right.$$

with *β_0 _*and *R*_0 _given by equations (B.3) and (B.2), respectively. In this case, the solution $\bar{y_{1}}$,

$$\bar{y_{1}}\;=\frac{\left(\mu +\gamma \right)\left(\mu +\delta +\alpha \right)}{\beta \left(\mu +\gamma +\delta +\alpha \right)}\left(R_{0}-1\right),$$

is positively defined for *R*_0 _> 1.

**Figure 1:**
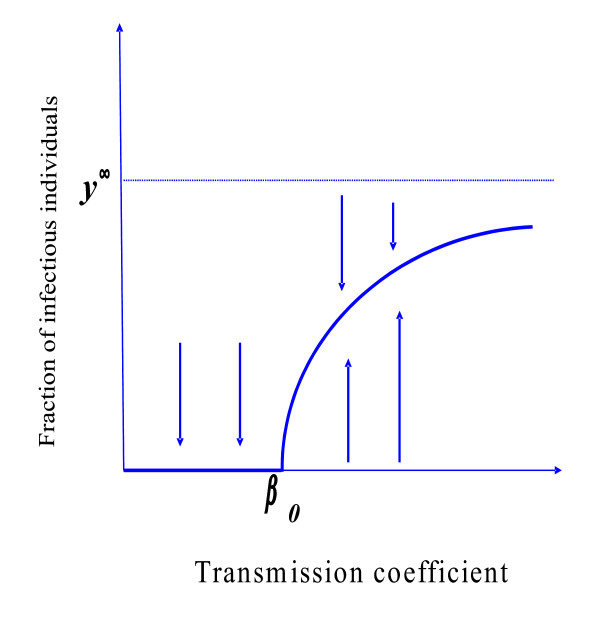
**The fraction of infectious individuals **$\bar{y}$ as function of transmission coefficient *β*, when *q *= 1. We present a qualitative bifurcation diagram in the case *γ≠0 *and *p *= 0.

Figure 1 shows the fraction of infectious individuals $\bar{y_{1}}$ as a function of the transmission coefficient *β*. For *β > β_0 _*the disease-free community is the unique steady state of the dynamical system. At *β = β_0 _*we have the trivial equilibrium $\bar{y_{1}}=0$ and, thereafter, for *β > β_0_*, we have a unique non-trivial equilibrium $\bar{y}$. This point increases with *β *to the asymptote $\underset{\beta \to \infty }{\lim}\bar{y_{1}}={\bar{y}}_{1}^{\infty }=\frac{\gamma }{\mu +\gamma +\delta +\alpha }$.

In the absence of the re-infection among recovered individuals, *q *= 0, we have

$$\bar{y_{0}}\;=\frac{\mu \left(\mu +\gamma \right)\left(\mu +\delta +\alpha \right)}{\beta \left[\mu \left(\mu +\gamma +\delta +\alpha \right)+\gamma \delta \right]}\left(R_{0}-1\right),$$

reaching the asymptote $\underset{\beta \to \infty }{\lim}\bar{y_{0}}={\bar{y}}_{0}^{\infty }=\frac{\mu \gamma }{\mu \left(\mu +\gamma +\delta +\alpha \right)+\gamma \delta }$. As expected, the case without re-infection presents lower incidence than that with re-infection [16]: $\bar{y_{1}}>\bar{y_{0}}$, and both cases have the same bifurcation value.

Let us make a brief remark about β_0_, the threshold of the transmission coefficient *β*, which is one of the main results originating from the mass action law. Substituting the threshold value β_0_, given by equation (B.3), into equation (B.2), we have

$$R_{0}=\frac{\beta }{\beta_{0}}=\frac{\gamma }{\left(\mu +\gamma \right)}\times \frac{\beta }{\left(\mu +\delta +\alpha \right)},$$

which gives the average number of infections resulting from one infectious individual (see [1] for details). However, the total contact rate can be expressed as β = β* N, where β* is the per-capita contact rate. Substituting *β *by β*N in the definition of *R*_*0*_, we can re-write it as

$$R_{0}=\frac{N}{N_{0}},$$

where *N*_*0*_, the critical (or threshold) size of the population, given by

(4) $$R_{0}=\frac{\left(\mu +\gamma \right)\left(\mu +\delta +\alpha \right)}{\gamma \beta^{*}},$$

is the minimum number of individuals required to trigger and to sustain an epidemic.

Let us suppose that a constant population size *N *is given. In this situation, *β *must be greater than the threshold contact rate *β*_*0 *_to result in an epidemic. Conversely, let us assume that the per-capita contact rate $\beta^{*}$ is given, but the population size varies. In this situation, an epidemic is triggered only when the threshold population size *N*_*0 *_is surpassed. Note that the critical population size *N*_*0 *_decreases as the per-capita contact rate β* increases.

### Modelling absence of natural flow to TB

Let us assess the influence of super-infection (*p *> 0) on the transmission of infection, when the latent period is very large (biologically γ → 0, but mathematically we consider *γ = 0*). We are dealing with the case where the infected individuals remain in the exposed class until they either catch multiple infections or die.

In the steady state of the system of equations (2), we have the trivial equilibrium point *P*^0 ^= (1,0,0,0), which is always stable, as shown in Appendix B.

With respect to the non-trivial equilibrium point, letting *γ = 0 *in equation (B.8) with $\underset{\gamma \to 0}{\lim}\beta_{0}\to \infty$, we present two special cases: *q *= 0 and *q *= 1.

When *q *= 1, we have zero or two positive equilibria, which are the roots of the polynomial $Q\left(\beta \bar{y}\right)$ given by equation (B.7), where the coefficients are

$$\left\{ \begin{array}{l} a_{2}=p\\ a_{1}=p\left(\beta_{1}-\beta \right)\\ a_{0}=\mu \left(\mu +\delta +\alpha \right)\;, \end{array}\right.$$

and *β*_*1 *_is, from equation (B.9), letting *γ = 0*,

$$\beta_{1}=\left(\mu +\delta +\alpha \right)\left(1+\frac{1}{p}\right)\;.$$

The polynomial $Q\left(\bar{y}\right)$ has two positive roots ${\bar{y}}_{1}^{+}$ and ${\bar{y}}_{1}^{-}$, with

$${\bar{y}}_{1}^{\pm }=\frac{\left(\beta -\beta_{1}\right)}{2\beta }\left[1\pm \sqrt{1-\frac{4\mu \left(\mu +\delta +\alpha \right)}{p{\left(\beta -\beta_{1}\right)}^{2}}}\right]\;,$$

when $\beta \;>\beta_{c}^{1}$, where $\beta_{c}^{1}$, from equation (B.13) with *y *= 0, is the turning value given by

$$\beta_{c}^{1}=\beta_{1}+\sqrt{\frac{4}{p}\mu \left(\mu +\delta +\alpha \right)}\;.$$

These positive roots collapse to a unique ${\bar{y}}_{1}^{*}$ given by

$${\bar{y}}_{1}^{*}=\frac{\sqrt{\frac{4}{p}\mu \left(\mu +\delta +\alpha \right)}}{2\left[\beta_{1}+\sqrt{\frac{4}{p}\mu \left(\mu +\delta +\alpha \right)}\right]}$$

at $\beta \;=\beta_{c}^{1}$. For $\beta \;<\beta_{c}^{1}$ there are no positive real roots.

**Figure 2:**
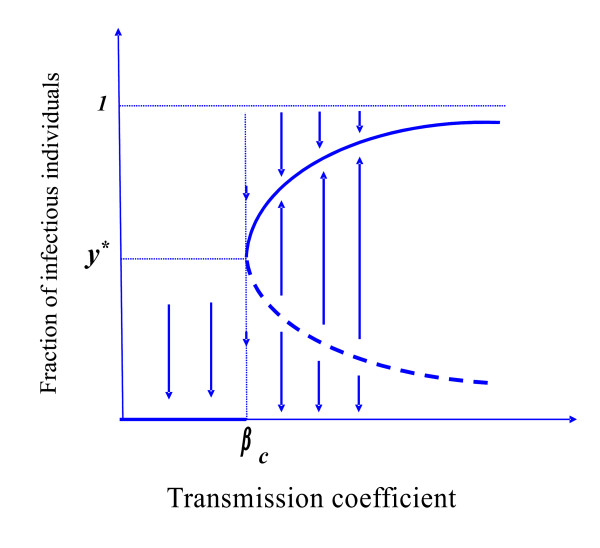
**The fraction of infectious individuals **$\bar{y}$ as function of transmission coefficient *β*, when *q *= 1. We present a qualitative bifurcation diagram in the case *γ = 0 *and *p*≠0.

Figure 2 shows the fraction of infectious individuals ${\bar{y}}_{1}^{\pm }$ as a function of the transmission coefficient *β*. For $\beta \;<\beta_{c}^{1}$ the disease-free equilibrium is a unique steady state of the dynamical system. At $\beta \;=\beta_{c}^{1}$, the turning value, there arises a collapsed non-trivial equilibrium ${\bar{y}}_{1}^{*}$, called the turning equilibrium point P^* ^[17], which is given by P* = (s*, e*, y*, z*). Thereafter, for $\beta \;>\beta_{c}^{1}$, two distinct branches of equilibrium values emerge from the same ${\bar{y}}_{1}^{*}$. Hence, $\beta_{c}^{1}$ is the threshold value since it separates the region where we have eradication of the disease ($\beta \;<\beta_{c}^{1}$) from the region where it becomes endemic ($\beta \;>\beta_{c}^{1}$). The large equilibrium ${\bar{y}}_{1}^{+}$ increases with *β*, reaching the asymptote $\underset{\beta \to \infty }{\lim}{\bar{y}}_{1}^{+}=1$, while the small equilibrium ${\bar{y}}_{1}^{-}$ decreases with *β*, reaching the asymptote $\underset{\beta \to \infty }{\lim}{\bar{y}}_{1}^{-}=0$.

Let us consider the interval $\beta \;>\beta_{c}^{1}$. In this interval we have, besides the stable equilibrium point *P^0^*, two other equilibrium points $P^{-}=\left({\bar{s}}_{1}^{-},{\bar{e}}_{1}^{-},{\bar{y}}_{1}^{-},{\bar{z}}_{1}^{-}\right)$ and $P^{-}=\left({\bar{s}}_{1}^{+},{\bar{e}}_{1}^{+},{\bar{y}}_{1}^{+},{\bar{z}}_{1}^{+}\right)$, which are represented, respectively, by the lower and upper branches of the curve in Figure 2. The unstable equilibrium point *P*^- ^is called the 'break-point' [17,15], which separates two attracting regions containing one of the equilibrium points *P*^0 ^and *P*^+^. In other words, there is a surface (or a frontier) separating two attracting basins generated by the coordinates of the equilibrium point *P*^-^, *e.g. *$f\left({\bar{s}}_{1}^{-},{\bar{e}}_{1}^{-},{\bar{y}}_{1}^{-},{\bar{z}}_{1}^{-}\right)=0$, such that one of the equilibrium points *P*^0 ^and *P*^+ ^is an attractor depending on the relative position of the initial conditions $G=\left(s_{0}\;,\;e_{0}\;,\;y_{0}\;,\;z_{0}\right)$ supplied to the dynamical system (2) with respect to the surface *f *[18]. The term 'break-point' was used by Macdonald to denote the critical level for successful introduction of infection in terms of an unstable equilibrium point. The 'break-point' appears because super-infection is essential for the onset of disease in the absence of natural flow to the disease. When the transmission coefficient is low, relatively many infectious individuals must be introduced to trigger an epidemic; however, this number decreases as *β *increases.

In the absence of the re-infection among recovered individuals, *q *= 0, we have for the polynomial $Q\left(\beta \bar{y}\right)$, given by equation (B.7), the coefficients

$$\left\{ \begin{array}{l} a_{2}=p\left(\mu +\delta \right)\\ a_{1}=p\mu \left(\beta_{1}-\beta \right)\\ a_{0}=\mu^{2}\left(\mu +\delta +\alpha \right)\;, \end{array}\right.$$

where *β*_1 _is the same as for the case *q *= 1. Hence, when $\beta \;>\beta_{c}^{0}$, where $\beta_{c}^{0}$ is

$$\beta_{c}^{0}=\beta_{1}+\sqrt{\frac{4}{p}\left(\mu +\delta \right)\left(\mu +\delta +\alpha \right)}\;,$$

we have two positive roots ${\bar{y}}_{+}^{0}$ and ${\bar{y}}_{-}^{0}$ given by

$${\bar{y}}_{0}^{\pm }=\frac{\left(\beta -\beta_{1}\right)\mu }{2\beta \left(\mu +\delta \right)}\left[1\pm \sqrt{1-\frac{4\left(\mu +\delta \right)\left(\mu +\delta +\alpha \right)}{p{\left(\beta -\beta_{1}\right)}^{2}}}\right]\;.$$

Note that at $\beta \;=\beta_{c}^{0}$ the positive roots collapse to a unique ${\bar{y}}_{0}^{*}$,

$${\bar{y}}_{0}^{*}=\frac{\mu \sqrt{\frac{4\;}{p}\;\left(\mu +\delta \right)\left(\mu +\delta +\alpha \right)\;}}{2\left(\mu +\delta \right)\sqrt{\beta_{1}+\left[\frac{4\;}{p}\;\left(\mu +\delta \right)\left(\mu +\delta +\alpha \right)\right]}}\;.$$

The large equilibrium ${\bar{y}}_{0}^{+}$ increases with *β*, reaching the asymptote $\underset{\beta \to \infty }{\lim}{\bar{y}}_{0}^{-}=\frac{\mu }{\mu +\delta }$, while the small equilibrium ${\bar{y}}_{-}^{0}$ decreases with β, reaching the asymptote $\underset{\beta \to \infty }{\lim}{\bar{y}}_{0}^{-}=0$. In comparison with the case *q *= 1, we have $\beta {\;}_{c}^{1}<\beta_{c}^{0}$, and ${\bar{y}}_{1}^{+}>{\bar{y}}_{0}^{+}$ and ${\bar{y}}_{1}^{-}<{\bar{y}}_{0}^{-}$ for every *β*, and ${\bar{y}}_{1}^{*}>{\bar{y}}_{0}^{*}$. This fact shows that re-infection acts: (1) to increase the incidence; (2) to diminish the region of attraction of the trivial equilibrium point; and (3) to decrease the turning value of the transmission coefficient.

Summarizing, when *γ = 0 *and *p > 0*, the bifurcation diagram shows that: (a) for $\beta \;<\beta_{c}^{q}$, *q *= 0,1, the trivial equilibrium *P*^0 ^is the unique attractor; and (b) for $\beta \;>\beta_{c}^{q}$, we have two basins of attraction containing the stable equilibrium points *P*^0 ^and *P*^+^, separated by a surface generated by the coordinates of the unstable equilibrium $P^{-}=\left({\bar{s}}_{i}^{-},{\bar{e}}_{i}^{-},{\bar{y}}_{i}^{-},{\bar{z}}_{i}^{-}\right),\;\;i=0,\;1$. The break-point *P*^- ^never assumes negative values.

### Model for TB transmission

When *p *= 0, the forward bifurcation is governed by the threshold *β*_0_. When *γ = 0*, we have the turning value $\beta_{c}^{q}$ and the 'break-point' *P*^- ^governing the dynamics, originating the hysteresis-like effect [19]. The dynamics of MTB transmission encompassing both super-infection and long latency are better understood as a combination of the previous results. We also take reinfection (*q*) into account, but analytical results are obtained for *q = 0 *and *q *= 1. We assumed that the 'fast' progress to the disease is due to super-infection (*p *> 0), while the 'slow' progress is due to a long period of time in the exposed class *(γ > 0*). Notice that the threshold transmission coefficient *β*_0_, given by equation (B.3), decreases when incubation rate *γ *increases: $\underset{\gamma \to 0}{\lim}\beta_{0}=\infty$ and $\underset{\gamma \to \infty }{\lim}\beta_{0}=\left(\mu +\delta +\alpha \right)$. If the time of natural flow from exposed to infectious class increases (*γ *decreases), the threshold $\beta_{0}$ increases and, as a consequence, the infection encounters more resistance to becoming established in a community (*β *must assume a high value in order to surpass *β*_0_).

In the previous two subsections, we showed particular sub-models. Here we use results from Appendix B, stressing that when: (a) γ>γ_+ _and (b) γ<γ_+ _and (c) *p*<*p*_0_, the dynamical behaviour is similar to that case without superinfection. Hence, we deal with the case γ<γ_+ _and *p*>*p*_0_.

**Figure 3:**
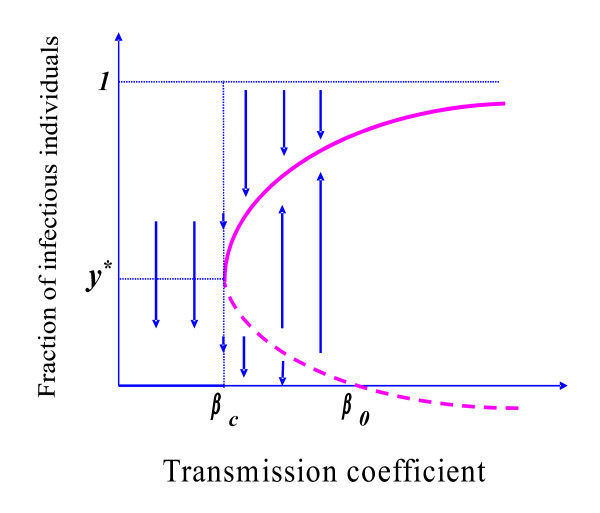
**The fraction of infectious individuals **$\bar{y}$ as function of transmission coefficient *β*, when *q *= 1. We present a qualitative bifurcation diagram in the case 0 *< γ < γ*_+ _and *p > p_0_*.

Let us consider *γ*<*γ*_+ _and *p*>*p*_0 _(the acquired immune response is not very strong). In this case, the polynomial $Q\left(\beta \bar{y}\right)$, given by equation (B.7), has in the range $\beta_{c}^{q}<\beta <\beta_{0}$ a large stable equilibrium ${\bar{y}}_{+}^{0}$ and a small unstable equilibrium ${\bar{y}}_{-}^{0}$. This behaviour accords with the result obtained with *γ = 0*. However, when *β *>*β*_0_, the very slow natural flow from exposed to infectious class affects the 'break-point'. Even when conditions γ<γ_+ _and *p*>*p*_0 _are satisfied, if the transmission coefficient surpasses the threshold value *β_0_*, then the value of the 'break-point' *P*^- ^becomes negative, and the unique positive solution is an attractor. Therefore, as expected, when *γ≠0 *and *R*_0 _> 1, we have only one positive solution. Figure 3 shows this behaviour.

The backward bifurcation diagram shown in Figure 3 is a combination of the diagrams shown in Figures 1 and 2. When $\gamma <\gamma_{+}$ but the immune response is low (*p *>*p*_0_), super-infection, which occurs during the incubation period (*γ*^-1^) and promotes a 'short-cut' to the onset of disease, is effectively an ally to supply enough infectious individuals to trigger an epidemic. When the transmission coefficient is small ($\beta \;<\beta_{c}^{q}$), super-infection does not matter because the number of infectious individuals is much lower than the critical number (see Discussion). But as *β *increases, more infectious individuals arise by natural flow from the exposed class and approach the critical number. The remaining infectious individuals, who become fewer with increasing *β*, are furnished by super-infection. For this reason the dynamical trajectories depend on the initial conditions and the 'break-point' decreases with increasing *β*. However, when *β *>*β*_*0*_, super-infection does not matter, because the natural flow from exposed to infectious class is sufficient to surpass the critical number. When the transmission coefficient surpasses the threshold value *β*_*0*_, the 'break-point' *P*^- ^becomes negative, meaning that the dynamical trajectories no longer depend on the initial conditions. Nevertheless, this behaviour is not observed when *p *<*p*_0 _(strong immune response), because the additional infectious individuals are not enough to attain the critical number and the epidemic fades away.

We present numerical results to illustrate the TB transmission model, using the values of the parameters given in Table 1, which are fixed unless otherwise stated. The value for the threshold transmission coefficient is *β_0 _= 5.2676 years*^-1^, from equation (B.3).

**Table 1:** The values assigned for the model's parameters.

Parameters	Values	Units
μ	0.016	*years*^-1^
α	0.01	*years*^-1^
γ	0.01	*years*^-1^
δ	2.0	*years*^-1^
β	4.9	*years*^-1^
*p*	0.8	--
*q*	1.0	--

From the values given in Table 1 we calculate, for *q *= 0: the critical parameter *β_1 _= 6.1335 years*^-1^, from equation (B.16), the critical proportion *P*_*0 *_= *1.014*, and the critical incubation rate *γ_+ _= 0.0099 years*^-1^, from equation (B.17). Note that for $\gamma >\gamma_{+}$, which implies *p*_0 _> 1, we have *β*_1 _>*β*_0_, for which reason $\beta_{c}^{0}$ and *R*_*p *_are not real numbers (see equation (B.18) for $\beta_{c}^{0}$). For *q *= 1 we have: the critical parameter *β_1 _= 4.5710 years*^-1^, from equation (B.9), the critical proportion *p*_*0 *_= *0.6281*, from equation (B.10), the critical incubation rate *γ_+ _= 0.01588 years*^-1^, from equation (B.12), the lower bound for the transmission coefficient $\beta_{c}^{1}=\text{4}\text{.7343 }years^{-1}$, from equation (B.13), and the turning value *R*_*p *_= 0.8988, from equation (B.15). In this case we have backward bifurcation, and we have ${\bar{y}}_{1}^{*}=0.01725$ from equation (B.14).

**Figure 4:**
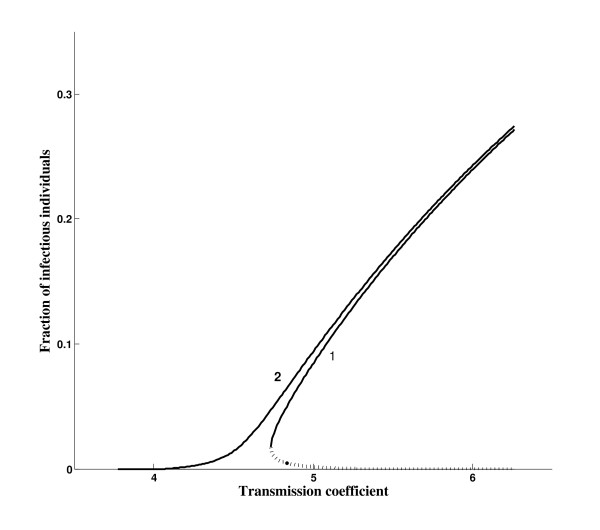
**The fraction of infectious individuals **$\bar{y}$**as function of transmission coefficient *β***. The curve on the right (labelled by 1) corresponds to the values given in Table 1 (resulting in *γ *<*γ*_+_); and for the curve on the left (labelled by 2), we changed only *γ, γ = 0.01588 years*^-1 ^(resulting in *γ *= *γ*_+_). In the curve representing the backward bifurcation, the solid line corresponds to the stable branch (${\bar{y}}^{+}$) and the dotted line to the unstable branch (${\bar{y}}^{-}$). Here we have *q *= 1 and *p *>*p*_0_. In this case backward bifurcation occurs over a narrow range *(*$\beta_{c}^{1}=4.7343$ and *β*_*0 *_= *5.2676 *both in *years*^-1^).

Figure 4 shows the equilibrium points (for *q *= 1), the solutions of the polynomial $Q\left(\beta \bar{y}\right)$ given by equation (B.7), as a function of the transmission coefficient *β*. The curve on the right (labelled 1) corresponds to the case *γ *<*γ*_+ _and *p *>*p*_*0*_, while the curve on the left (labelled 2) to *γ *= *γ*_+ _(at *γ *= *γ*_+ _we have *p*_0 _= 1 and *β*_0 _= *4.0679 years*^-1^). At *γ *= *γ*_+_, and above this critical value, the backward bifurcation disappears. We observe hysteresis in the backward bifurcation diagram (curve 1): *β *is decreased below the threshold value *β*_0 _but disease levels do not diminish until *β *<*β *_*c*_.

The bifurcation diagram shown in Figure 4 reveals some important features with respect to backward bifurcation, which occurs when *γ *<*γ*_+ _(and *p *>*p*_0_). However, increasing only the parameter γ (to enhance this behaviour, we let *γ *= *γ*_+_), the fraction of infectious individuals $\left(\bar{y_{1}}\right)$ is greater than the large value (${\bar{\;y}}_{1}^{+}$) corresponding to the case *γ *<*γ*_+_. As we have pointed out, when γ increases, *β*_0 _decreases, so *R*_0 _increases for fixed *β*. For this reason the curve with respect to the number of infectious individuals corresponding to a fixed *γ*, say $\bar{\gamma }$, always envelops all curves obtained with γ lower than $\bar{\gamma }$, when all other parameters are fixed.

Comparing results obtained from *q = 0 *and *q *= 1, we conclude that there is a critical value for *q*, named *q*_*c*_, below which we have no backward bifurcation. Let us determine this value. For each *q*, the equation $Q^{*}\left(\beta \;\bar{y,\;}\right)$ given by (B.5) with the coefficients given by equation (B.6), is such that $a_{3}^{*}$ does not depend on *β*, while $a_{2}^{*},a_{1}^{*}$ and $a_{0}^{*}$ do. Hence, we will write it as $Q^{*}\left(\bar{y,\;}\beta \;\right)$. When *γ *<*γ*_+ _and *p *>*p*_0_, at $\beta \;=\beta_{c}^{q}$ we have a single positive solution $y_{q}^{*}$, from which two positive solutions arise in the range $\beta_{c}^{q}<\beta \;<\beta_{0}$. According to Figure 4 (curve 1), we observe that

$$\frac{d\beta }{dy}=0$$

at $\beta \;=\beta_{c}^{q}$. To determine $\beta_{c}^{q}$, we differentiate both sides of the equation $Q^{*}\left(\bar{y,\;}\beta \;\right)=0$ by $\bar{y}$, resulting in

$$\frac{d}{d\bar{y}}\;Q^{*}\left(\bar{y},\beta \right)=0,$$

or

$$\frac{d}{d\bar{y}}\;Q^{*}\left(\bar{y},\beta \right)=\frac{\partial }{\partial \bar{y}}Q^{*}\left(\bar{y},\beta \right)+\frac{\partial }{\partial \beta }Q^{*}\left(\bar{y},\beta \right)\frac{d\beta }{d\bar{y}}=0\;.$$

But at $\beta \;=\beta_{c}^{q}$, we have $\frac{d\beta }{d\bar{y}}\;=0$, so $\frac{\partial }{\partial \beta }Q^{*}\left({\bar{y}}_{q}^{*},\beta_{c}^{q}\right)=0$. We must search for a positive solution of the system

(5) $$\left\{ \begin{array}{l} a_{3}^{*}{\left(\beta \bar{y}\right)}^{3}+a_{2}^{*}{\left(\beta \bar{y}\right)}^{2}+a_{1}^{*}\left(\beta \bar{y}\right)+a_{0}^{*}=0\\ 3a_{3}^{*}{\left(\beta \bar{y}\right)}^{2}+2a_{2}^{*}\left(\beta \bar{y}\right)+a_{1}^{*}=0 \end{array}\right.$$

in terms of $\left(\beta \;,\bar{y}\right)$. The solution is $\left(\beta_{c}^{q}\;,{\bar{y}}_{q}^{*}\right)$.

When *q *assumes its positive critical value, *q*_*c*_, we must have $\beta_{c}^{q}=\beta_{0}$ and ${\bar{y}}_{q_{c}}^{*}=0$, and the algebraic system (5) becomes $a_{0}^{*}\left(\beta \right)=0$ and $a_{1}^{*}\left(\beta \right)=0$. At $\beta =\beta_{c}^{q_{{}_{c}}}=\beta_{0}$, we have $a_{0}^{*}\left(\beta_{0}\right)=0$, and *q*_*c *_can be found from $a_{1}^{*}\left(\beta_{0}\right)=0$, that is,

$$q_{c}=\frac{\gamma \left[\left(\mu +\gamma \right)\left(\mu +\gamma \right)+\mu \alpha \right]-p\mu^{2}\left(\mu +\delta +\alpha \right)}{\delta \gamma^{2}}\;.$$

Using the values of the parameters given in Table 1, we obtain *q*_*c *_= *0.5542*. Therefore, for $0\leq q\leq q_{c}$, the backward bifurcation disappears. Additionally, we can determine the value of γ, say *γ*_*min*_, such that *q*_*c *_= 0. Again, using the values of the parameters given in Table 1, we obtain *γ*_*min *_= *0.008405 years*_-1_. Hence, if *γ *<*γ*_*min*_, we have *q*_*c *_*< 0 *and backward bifurcation exists for all values of *q*. When *γ *= 0.008405 *years*_-1_, lower than the value given in Table 1, we have *β*_*0 *_= *5.8828 years*_-1_. In this case, we found $\beta_{c}^{0}=\beta_{0}$ and *q*_*c *_= 0, resulting in ${\bar{y}}_{0}^{*}=0$. When *q *= 1, we have *β*_*1 *_= *4.569 years*^-1^, *p*_*0 *_= *0.5275*, *R*_*p *_*= 0.8132*, and ${\bar{y}}_{1}^{*}=0.0225$.

**Figure 5:**
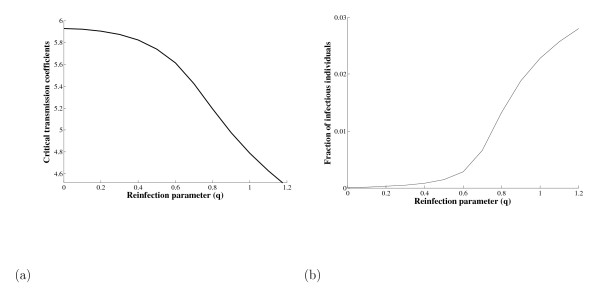
We show the critical transmission coefficient $\beta_{c}^{q}$ (a) and ${\bar{y}}_{q}^{*}$ (b) as a function of *q*. Using the values of the parameters given in Table 1, except *γ = 0.008405 years*^-1^, we have *q*_*c *_= 0, and ${\bar{y}}_{0}^{*}$. In this set of values the backward bifurcation exists for all *q*.

Considering the values given in Table 1, except *γ = 0.008405 years*^-1^, let us obtain the solution $\left(\beta_{c}^{q},{\bar{y}}_{q}^{*}\right)$ for each *q*. In Figures 5.a and 5.b we show, respectively, $\beta_{c}^{q}$ and ${\bar{y}}_{q}^{*}$ as functions of *q*. We apply the Newton-Raphson method to solve the algebraic equation (5). As an initial guess to solve the nonlinear system, we used previously calculated values at *q *= 0: $\beta_{c}^{0}=\beta^{0}=5.8828\;years^{-1}$ and ${\bar{y}}_{0}^{*}=0$. As *q *increases, $\beta_{c}^{q}$ decreases and ${\bar{y}}_{q}^{*}$ increases. Re-infection enlarges the range of *β *in which backward bifurcation in may occur.

Let us change only the value of the incubation rate in Table 1 obtained according to the following reasoning. Let us assume that the probability of a latently-infected person progressing to TB at age *a *follows an exponential distribution, or $p=1-e^{-\gamma a}$ (for the sake of simplicity, we assume primary infection at birth). If we assume that the probability of endogenous reactivation at life expectancy (for instance, *a *= 100 *years*) is 10%, then we estimate *γ = 0.0011 years*^-1 ^(for 5%, we have *γ = 0.00051 years*^-1^). Hence, let us set *γ = 0.001 years*^-1^, lower than *γ*_*min*_. In this case we have *β*_0 _=*34.442 years*^-1^. The new evaluations for *q = 0 *are: *β*_*1 *_= *4.716 years*^-1^, *p*_*0 *_= *0.0664*, *γ*_+ _= *0.0099 years*^-1^, $\beta_{c}^{0}=5.1107\;years^{-1}$, *R*_*p *_= *0.1484*, and ${\bar{y}}_{0}^{*}=0.03862$. For *q *= 1, we have: *β*_*1 *_= *4.560 years*^-1^, *p*_*0 *_= *0.0625*, *γ*_+ _= *0.01588 years*^-1^, $\beta_{c}^{1}=4.9438\;years^{-1}$, *R*_p _= *0.1435*, and ${\bar{y}}_{1}^{*}=0.03884$. In this set of parameter values, we have *q*_*c *_= *-190.2*, and backward bifurcation occurs for all values of *q*. In the best scenario (*q *= 0), we have *R*_p _= *0.1484*, showing an extremely dangerous epidemiological situation promoted by both super-infection and reinfection (the threshold *β*_*0 *_is very high).

Let us compare the results obtained using the values given in Table 1 with the set of values at which we decrease only the value of the incubation rate tenfold, that is, *γ = 0.001 years*^-1^. We obtain: *β*_0 _= *34.442 years*^-1^, increasing around six and half times; *p*_0 _= *0.0664 *(when *q = 0*), decreasing around fifteen times; and $\beta_{c}^{1}$ (for *q *= 1) varies little, but *R*_*p *_decreases more than six times. Increasing the incubation period diminishes the risk of TB transmission, but the 'short-cut' to TB promoted by super-infection makes the transmission of MTB practicable for some range of values of the transmission coefficient (*β*_0 _= *5.2676 years*^-1 ^corresponding to Table 1, and $\beta_{c}^{0}=5.1107\;years^{-1}$ in this case with *q = 0*).

**Figure 6:**
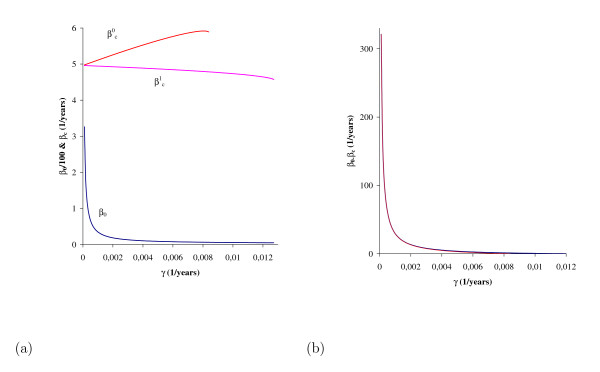
**The threshold (*β***_***0***_**) and lower bound ($\beta_{c}^{q}$, for *q = *0 and 1) transmission coefficients as a function of the incubation rate γ, using values given in Table 1**. *β*_*0 *_(multiplied by a factor 100) and $\beta_{c}^{1}$ are decreasing functions, while $\beta_{c}^{0}$ is an increasing function, with $\beta_{0}>\beta_{c}^{0}>\beta_{c}^{1}$. When *q *= 1, they assume the same value ($\beta_{0}=\beta_{c}^{1}=\text{4}\text{.55 }years^{-1}$) at *γ = 0.0128 years*^-1^, and for *q *= 0, they assume the same value ($\beta_{0}=\beta_{c}^{0}=\text{5}\text{.891 }years^{-1}$) at *γ = 0.00838 years*^-1 ^(a). At a given γ, the difference between *β*_*0 *_and $\beta_{c}^{1}$ (or $\beta_{c}^{0}$, which is practically the same) corresponds to the range of *β *at which two positive solutions are found (b).

Backward bifurcation occurs in the interval $\beta_{c}^{q}<\beta <\beta_{0}$. *β*_*0 *_does not depend on *p *and *q*, but $\beta_{c}^{q}$ does. Let us study how the lower bound ($\beta_{c}^{q}$) and the length ($\beta_{0}<\beta_{c}^{q}$) of occurrence of backward bifurcation depend on the incubation rate γ. In Figure 6 we illustrate this using the values given in Table 1. For *q *= 0 and *q *= 1 we calculate the lower bound $\beta_{c}^{q}$, and the threshold that does not depend on *q*. When *q *= 0, we have the least likelihood of backward bifurcation: (a) for this reason we have $\beta_{c}^{0}>\beta_{c}^{1}$ for each γ, and (b) we have the lowest value for γ, say *γ*_*min*_, above which backward bifurcation disappears and forward bifurcation dominates the dynamics (Figure 6.a). Figure 6.b shows that the range of *β *at which we have two positive solutions (backward bifurcation) increases quickly for *γ = 0.002 years*^-1^, and blows up for *γ< 0.001 years*^-1^. The lowest value above which the backward bifurcation is substituted by forward is *γ*_*min *_= 0.0128 *years*^-1 ^for *q *= 1 ($\beta_{0}=\beta_{c}^{1}=4.55\;\;years^{-1}$), and *γ*_*min *_= 0.00838 *years*^-1 ^for *q *= 0 ($\beta_{0}=\beta_{c}^{0}=5.891\;\;years^{-1}$).

**Figure 7:**
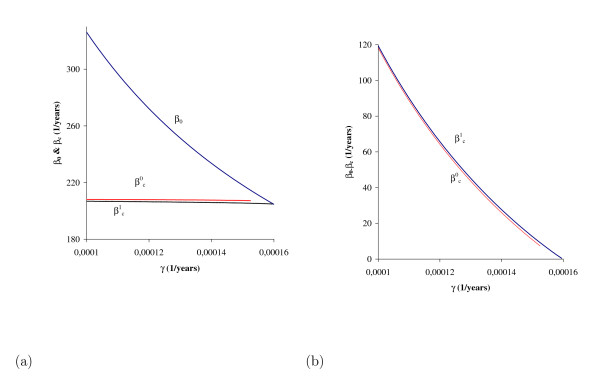
**The threshold (*β***_***0***_**) and lower bound ($\beta_{c}^{q}$, for *q *= 0 and 1) transmission coefficients as a function of the incubation rate γ, using *p *= 0.01; all other values are those given in Table 1**. *β*_*0*_, $\beta_{c}^{0}$ and $\beta_{c}^{1}$ are decreasing functions, with $\beta_{0}>\beta_{c}^{0}>\beta_{c}^{1}$. When *q *= 1, they assume the same value ($\beta_{0}=\beta_{c}^{1}$ = 205 *years*^-1^) at *γ = 0.0001595 years*^-1^, and for *q *= 0, they assume the same value ($\beta_{0}=\beta_{c}^{0}$ = 207 *years*^-1^) at *γ = 0.000158 years*^-1 ^(a). At a given γ, the difference between *β*_*0 *_and $\beta_{c}^{1}$ (or $\beta_{c}^{0}$, which is practically the same) corresponds to the range of β in which two positive solutions are found (b).

In Figure 7 we illustrate the backward bifurcation when the immune system mounts a strong response. We use the values given in Table 1, except *p *= 0.01. The backward bifurcation occurs for very low incubation rate, and the lower bound of the transmission coefficient ($\beta_{c}^{q}$) is practically constant but situated at a higher value (200 *years*^-1^). This value is more than approximately 40 times the lower bound observed in the previous case (Figure 6.a). Once eradication of TB is achieved when $\beta <\beta_{c}^{q}$, a strong immune response, by administrating an appropriate stimulus to immune system, can easily eradicate MTB transmission. The lowest value above which the backward bifurcation is substituted by forward is *γ*_*min *_= 0.0001595 *years*^-1 ^for *q *= 1 ($\beta_{0}=\beta_{c}^{1}=205\;\;years^{-1}$), and *γ*_*min *_= 0.0001595 *years*^-1 ^for *q *= 0 ($\beta_{0}=\beta_{c}^{1}=207\;\;years^{-1}$).

**Figure 8:**
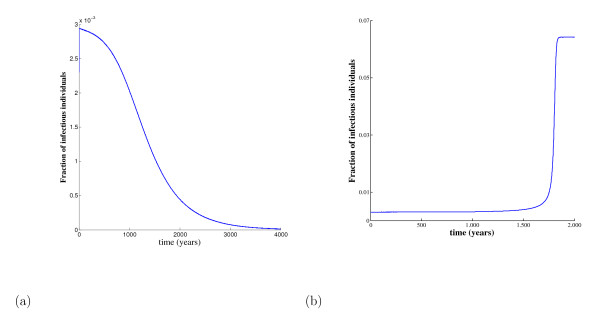
**The dynamical trajectories using values given in Table 1**. In (a) the initial conditions supplied are $G=\left({\bar{s}}_{1}^{-},{\bar{e}}_{1}^{-},0.999\times {\bar{y}}_{1}^{-},{\bar{z}}_{1}^{-}\right)$; and in (b), $G=\left({\bar{s}}_{1}^{-},{\bar{e}}_{1}^{-},1.001\times {\bar{y}}_{1}^{-},{\bar{z}}_{1}^{-}\right)$. In the former case, the initial conditions are contained in the region of attraction of *P*^0^, while in the latter, *P*^+^. Here we have *q *= 1, *γ < γ_+_, p > p_0 _*and *β *>*β_0_*.

Figure 8 shows the dynamical trajectories considering the values given in Table 1 ($\beta_{1}<\beta_{c}^{1}<\beta <\beta_{0}$). Figures 8.a and 8.b illustrate the case in which the dynamical trajectories are well defined disregarding the initial conditions. In this case, the initial conditions ($s_{0}={\bar{s}}_{1}^{-},\;\;e_{0}={\bar{e}}_{1}^{-},\;y_{0}=\left(1+\varepsilon \right){\bar{y}}_{1}^{-},\;\;\;z_{0}={\bar{z}}_{1}^{-}$, where ε = 0.001) deviate slightly from the unstable non-trivial equilibrium, which has coordinates *P*^- ^= (0.5236,0.2786,0.00298,0.1949)and divides two attracting regions. From Figure 4, it is easy to conclude, before numerical simulation, that the trajectories achieve a non-trivial equilibrium point *P*^+ ^if $y_{0}=\left(1-\varepsilon \right){\bar{y}}_{1}^{-}$ and a trivial equilibrium *P^0 ^*otherwise. However, if the initial conditions deviate markdly from *P^-^*, we cannot identify the attracting point unless a numerical simulation is performed. In general, we have a boundary formed by the coordinates of the break-point, or a surface satisfying the equation $f\left({\bar{s}}_{1}^{-},{\bar{e}}_{1}^{-},{\bar{y}}_{1}^{-},{\bar{z}}_{1}^{-}\right)=0$, that divides two attracting regions containing *P*^0 ^and *P*^+^. Hence, in special cases, such as the example shown in Figure 8, we can predict the outcome, which is not the case for general initial conditions *G *= (*s_0_, e_0_, y_0_, z_0_*) supplied to the dynamical system (2). The dependency on initial conditions disappears when $\beta_{1}<\beta_{c}^{1}$ (the attractor is the trivial equilibrium point *P^0^*) and *β *<*β_0 _*(the attractor is the unique *P*^+^).

Figure 8 was obtained using the set of values given in Table 1. In this case we have *R_0 _= 0.93*, lower than one but greater than *R_p _= 0.8999*, which is the reason for presenting trajectories depending on the initial conditions. Moreover, the initial condition for infectious persons *y*_0 _is 0.002977 (Figure 8.a) or 0.002983 (Figure 8.b), which is lower than ${\bar{y}}_{1}^{*}=0.01725$ (at *β = β_1_*). This set of initial conditions showed a very long time delay before the stable equilibrium point was achieved (that is, the plateau of the curve), in which case constant population size is not a good approximation. However, using the same initial conditions, and changing only the transmission coefficient yielding *R_0 _= 2 (β = 10.535 years*^-1^), the equilibrium point (plateau of the curve) is achieved earlier, at around 4.5 *years*, and for *R_0 _= 5 (β = 26.338 years*^-1^), at 1.2 *years *(figures not shown).

Figure 8 was generated for a sufficiently weak immune response. If we change only the value of *p *in Table 1, such that it is diminished below its critical *p*_0_, *p *= 0.5, the attracting region contains the trivial equilibrium point *P*_0_, independently of the initial conditions (figure not shown). In this case we do not have the backward bifurcation. On the other hand, if we change only the value of the transmission coefficient in Table 1, so as to surpass the threshold value, i.e. *β = 6.*0 *years*^-1 ^(*β > β_0_*), we have only one attracting region and, independently of the initial conditions, the dynamical system goes to the asymptotic equilibrium *P*^+ ^(figure not shown). When the transmission coefficient exceeds its critical value the attracting region of *P*^0 ^disappears (the 'break-point' *P*^- ^becomes negative), except when the initial conditions are *G *= (1,0,0,0).

Summarizing, forward bifurcation generally predominates in the analysis of the system of equations (2). However, when the natural progression of the infection is very slow and the rate of super-infection is high, we observe the hysteresis effect (backward bifurcation). Additionally, the initial conditions supplied to the dynamical system affect the trajectories only in the range $\beta_{c}^{1}<\beta <\beta_{0}$. As we have pointed out (in the case *q = 1*, absence of immune response), when $\gamma <\gamma_{+}$ (very slow onset of disease) and *p > p*_0 _(high rate of super-infection owing to weak immune response), we have two positive solutions in the interval $\beta_{c}^{q}<\beta <\beta_{0}$. Another important parameter is reinfection. When the immune response enhances the response against MTB among cured persons, there is a critical immune response, *q_c_*, below which the backward bifurcation disappears (*q < q_c_*). Hence, the general conditions for backward bifurcation are: (1) $\gamma <\gamma_{+}$ (long period of latency); (2) *p > p_0 _*(weak immune protection); and (3) *q > q_c _*(weak immunological memory). Note that *q_c _*can assume a zero value depending on the values assigned to the model's parameters. As we have shown above, when *γ = 0.008405 years*^-1^, lower than the value given in Table 1, $\beta_{c}^{q}=\beta_{0}$ and ${\bar{y}}_{0}^{*}=0$ because *q_c _= 0*, implying that the backward bifurcation always occurs for all ranges of *q*.

## Discussion

With respect to the model described by system (2), Lipsitch and Murray [20] claimed that the existence of multiple equilibria depends on unrealistic assumptions about the epidemiology of TB. They argue that, if (1) the probability that a contact between an infectious person and a susceptible person will lead to disease is $\beta \frac{\mu }{\mu +\gamma }$, and (2) the corresponding expression is *βp *for the contact between an infectious person and a latently infected person, then $p<\frac{\gamma }{\mu +\gamma }$. The reason behind this is that latent infection provides some immunity to reinfection. However, because $p_{0}>\frac{\gamma }{\mu +\gamma }$, the condition *p > p*_0 _implies $p>\frac{\gamma }{\mu +\gamma }$, a contradiction.

Note that the model described by system (2) is treated as an approximation of the general model given by system (1), which eliminates the unrealistic assumptions about the epidemiology of TB pointed out in [20]. The approximations to simplify the general system are the following. When *s << e *and *z << e*, which is true for γ~0 and *β >> 1*, we have p'βys+q'βyz<<pβye. In addition, if we deal with the limiting conditions p'<<1 and q'<<1, then (supposing the latter approximation is corroborated) (1+p')βys≈βys and (q+q')βyz≈qβyz, remembering that q'<q and *q *can exceed unity. The above suppositions are reasonable, if, for instance, *γ = 0.001 years*^-1^, in which case we obtained *β_0 _= 34.442 years^-1 ^*and *p_0 _= 0.0664*, and we can choose sufficiently large β and small *p *(remembering that *p < p'*). Moreover, we showed that backward bifurcation exists for all values of *q *for that value of *γ*. For *γ = 0.0001 years*^-1 ^(corresponding to 1% of endogenous reactivation of TB at *a = 100 years*) and *q *= 1 we obtain *β_0 _= 326.186 years^-1 ^*and *p_0 _= 0.00625*.

In developing countries, the above assumptions are quite valid. Let us understand that system (2) is an approximation of system (1) when primary TB and relapse to TB of cured individuals are negligible in comparison with super-infection. Hence, the unrealistic system (2) provides us with approximate results of biologically feasible modelling, and our results must be interpreted with caution.

The so-called backward bifurcation occurs over a very narrow range of incubation rate γ, that is, $\gamma <\gamma_{+}$, with $\gamma_{+}<\mu$. Additionally, we must have high levels of super-infection (owing to a weak immune response, satisfying *p > p*_0_) and reinfection (owing to a waning immunological memory, satisfying *q > q_c_*). The main aspects of backward bifurcation are (i) the dependency of the trajectories on the initial conditions supplied to the dynamical system $\beta_{c}^{q}<\beta <\beta_{0}$, and (ii) the lack of positive equilibrium for $\beta <\beta_{c}^{q}$, with $\beta_{c}^{q}<\beta_{0}$. However, the trajectories of the dynamical system do not depend on the initial conditions when the threshold transmission coefficient β is above the threshold *β_0_*: for *β ≥ β_0_*, the unstable branch assumes negative values. In all other cases, that is, (i) *γ < γ_+ _*and *p ≤ p_0 _*and (ii) *γ ≥ γ_+_*, we observe a forward bifurcation at *R*_0 _= 1.

With respect to *γ*_+_, it seems natural that one of the conditions necessary to yield backward bifurcation is *γ < γ_+_*. When *γ > μ*, or *γ^-1 ^< μ^-1^*, the onset of disease occurs during the average survival time of humans and, as a consequence, infectious individuals accumulate because of the natural history of disease, for which reason super-infection only increases the incidence, and the dynamics is ruled only by *β_0 _*(or *R_0_*), the threshold value. However, if an infectious disease presents a very long period of incubation, larger than the average survival time of the host (*μ*^-1^), then it seems reasonable that super-infection changes the dynamics: the dynamical trajectories depend on the initial conditions for low values of the transmission coefficient relative to the critical value *β_0_*. Hence super-infection acts as a 'short cut' to increase the number of infectious individuals and, when the critical number is surpassed, an epidemic is triggered at high level (hysteresis).

Let us understand the role of the initial conditions supplied to the dynamical system in the range $\beta_{c}^{q}<\beta <\beta_{0}$, for *γ < γ_+ _*and *p > p_0_*.

In a primary infection, low transmission rate (we are considering that $\beta_{c}^{q}<\beta <\beta_{0}$, that is, *R*_0 _< 1) implies that a small number of susceptible individuals are transferred to the exposed class. In the absence of super-infection, the number of infectives is not sufficient to maintain the disease. The threshold theory establishes that the disease fades away regardless of the number of infectious (or latent) individuals introduced in the community because *β *is below the critical level (*β_0_*) to trigger and maintain an epidemic. Notice that the first infection has as target all the susceptible individuals, while the second infection needs to target only the exposed individuals. However, super-infection among individuals dammed in the exposed class increases the number of infectious individuals because of the 'short cut' to onset of disease. For this reason, if a few infectious individuals (*y*_0_) are introduced into a community free of disease, so that it is above the critical number given by the equation (4), then an epidemic will be triggered and a long-term level of epidemic will be maintained. The is possible because the additional increase in the number of infectious individuals due to super-infection is essential to surpass the critical number, which is unreachable by natural flow from the exposed to infective class alone. For this reason the trajectories of the dynamical system depend on the initial conditions supplied to it. Notice that the critical number of infectious individuals being introduced into a community decreases as the transmission coefficient increases, and, when *β ≥ β_0_*, the natural flow from the exposed to infective class is sufficient to yield a number infectious individuals above the critical value.

The occurrence of backward bifurcation is situated in a very restrictive range of the incubation period. This period must exceed the human life-span, in which case the number of individuals with TB disease must be very low. However, according to Figure 4, lowering the incubation period (*γ*^-1^) is more dangerous than the behaviour due to super-infection: the increase in *γ *decreases *β_0 _*(*γ *from 0.0001 to 0.01 results in *β_0 _*from 326.186 to 5.2676 and $\beta_{c}^{1}$ from 4.9593 to 4.7343, all in *years*) and the curve relating to forward bifurcation envelops the curve corresponding to backward bifurcation. Notice that $\beta_{c}^{1}$ is quite unchanged, while *β_0 _*is decreased drastically. However, we must be aware of the maintenance of TB (in a very low incidence) even when the transmission coefficient is lower than its threshold value. The increasing trend in the world of diabetes, which induces moderately immunocompromising conditions [21], can change TB incidence among elders.

The increased incidence of AIDS has led to the resurgence of TB in regions where this disease was considered eradicated. MTB infection is now considered as an indicator of HIV infection [22,23], and TB can be considered the main opportunistic disease for AIDS. However, in developing countries, owing to the endemic character of TB [24], there is no well established correlation between AIDS and TB.

In many developed countries, TB transmission, which was considered controlled until the advent of AIDS, has re-emerged [6]. One explanation is the shortening of the incubation period due to immunosuppression as a consequence of AIDS. According to this point of view, when γ is increased, the threshold transmission coefficient *β*_0 _is decreased, according to equation (B.3). If the transmission coefficient *β *is low, then lowering *β_0 _*can be sufficient to ensure that the basic reproduction ratio *R_0 _*is greater than one. Hence, we expect that TB should be maintained at low prevalence. When the onset of TB due to AIDS does not explain the epidemiological findings fully, in this case super-infection [25] should be an agent enabling a 'short cut' to the quick onset of TB disease. Let us consider developed countries where TB is controlled, and assume that *γ < γ*_+_. If we consider that *γ *is increased due to AIDS, but is not sufficient to decrease *β_0 _*below *β*, as we did before, then another way to explain the re-emergence of AIDS is to evoke super-infection acting as a 'short cut' to the onset of TB. In this situation, if AIDS is able to generate sufficient TB diseased individuals, then even at a low transmission level, but in the range $\left[\beta_{c}^{q},\;\beta_{0}\right]$, the disease must be maintained at endemic level, according to the backward bifurcation. We stress that re-infection decreases $\beta_{c}^{q}$ (Figure 5.a), which is another source for the re-emergence of TB.

Let us consider the parameters values given in Table 1. In Table 2 we present the special values of the transmission coefficients (*β_0 _*and $\beta_{c}^{q}$) considering four values of *γ*. We also calculated for a strong immune response, that is, *p *= 0.01. In this case, when *γ = 0.00016 years*^-1^, we have *β_0 _= 204.626 years*^-1 ^and a slightly higher *β_1 _= 204.642 years*^-1^, hence backward bifurcation disappears.

**Table 2:** For different values of *γ*, we present the threshold (*β*_*0*_) and lower bound ($\beta_{c}^{q}$, for *q *= 0 and 1) of the transmission coefficients (all in *years*^-1^).

		*p *= 0.80				*p *= 0.01			
*γ*	*β_0_*	$\beta_{c}^{0}$	$\beta_{c}^{1}$	$\beta_{0}-\beta_{c}^{0}$	$\beta_{0}-\beta_{c}^{1}$	$\beta_{c}^{0}$	$\beta_{c}^{1}$	$\beta_{0}-\beta_{c}^{0}$	$\beta_{0}-\beta_{c}^{1}$
0.01	5.2676	--	4.7343	--	0.5333	--	--	--	--
0.001	34.442	5.1107	4.9438	29.3313	29.4982	--	--	--	--
0.0001	326.19	4.9763	4.9594	321.2137	321.2306	208.07	208.82	118.12	119.37
0.00001	3243.6	4.9626	4.9609	3238.6374	3238.6391	208.24	208.11	3035.4	3035.5

In the last two columns we present the range over which backward bifurcation occurs ($\beta_{0}-\beta_{c}^{q}$). For γ = 0.001 *years*^-1^, we have no backward bifurcation for *q *= 0 ($\beta_{c}^{0}$ does not exist). We considered *p *= 0.8 and *p *= 0.01, and the values of other parameters are those given in Table 1.

**Figure 9:**
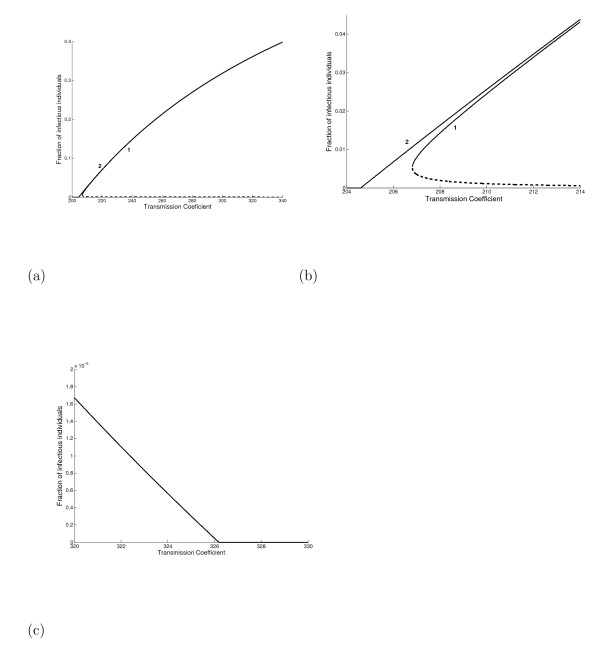
**The bifurcation diagram (a) in a strong immune response, that is, *p *= 0.01, for two values of γ (in *years*^-1^): 0.0001 (backward bifurcation, labelled 1) and *0.00016 *(forward bifurcation, with *β***_***0***_** = 204.626 $years^{-1}$, labelled 2)**. We give, in (b) and (c) respectively, a zoom near the lower bound ($\beta_{c}^{1}=\text{208}\text{.82 }years^{-1}$) and threshold (*β*_*0*_* = 326.19 years*^-1^) transmission coefficients with respect to the backward bifurcation.

In Figure 9 we present the bifurcation diagram in the case of a strong immune response, that is, *p *= 0.01. We consider two values of *γ *(in *years*^-1^): 0.0001 (backward bifurcation) and 0.00016 (forward bifurcation, with *β_0 _= 204.626 years*^-1^, and below this value the negative values were changed to zero), shown in Figure 9.a. In Figures 9.b and 9.c we zoom near, respectively, the lower bound ($\beta_{c}^{1}=208.82years^{-1}$) and threshold (*β_0 _= 326.19 years*^-1^, and above this value the negative values were changed to zero) transmission coefficients with respect to the backward bifurcation. When *β = 326.19 years*^-1^, corresponding to the threshold in the backward bifurcation, in the case of the forward bifurcation we have *R_0 _= 1.59*. At this value of *R*_0 _we have a nearly 37.6% prevalence of active TB. Notice that the curve of forward bifurcation envelops, but is practically coincident with, the stable branch (large equilibrium point) of the backward bifurcation. On the other hand, the unstable branch (small equilibrium point) of the backward bifurcation is situated near zero prevalence. The trivial equilibrium is unstable in the forward but stable (below *β*_0_) in the backward bifurcation. When *p = 0.8 *(see Figure 4), a weak immune response, we have for two values of *γ *(in *years*^-1^): 0.01 (backward bifurcation) and 0.0129 (forward bifurcation, with *β_0 _= 4.53887 years*^-1^). When *β = 5.2676 years*^-1^, corresponding to the threshold in the backward bifurcation, in the case of the forward bifurcation we have *R_0 _= 1.16*. However, when *β = 326.186 years*^-1^, corresponding to the threshold in the backward bifurcation with *γ = 0.0001 years*^-1^, in the case of the forward bifurcation with *γ = 0.0129 years*^-1 ^we have *R_0 _= 71.87*.

The immune response is affected by many factors, among them nutritional status, health conditions and genetic factors. As we have shown in Figures 4 (weak immune response) and 9 (strong immune response), a weakening of the immune response facilitates the appearance of backward bifurcation in the sense of shortening the incubation period (see Table 2). Moreover, a shortening of the incubation period due to immunosuppression, for instance, tends to eliminate this kind of bifurcation. However, backward bifurcation is not a catastrophic behaviour because of these major aspects: a small increase in the incubation rate results in forward bifurcation, which envelops the curve of backward bifurcation, and the unstable branch (small positive solutions) is in general so low that is confounded with the zero value.

## Conclusions

The model proposed here is an approximation of the general model that takes into account primary TB, according to system (1). A simplified model taking into account a very long latent period and super-infection in the exposed class (MTB positive) and reinfection of recovered individuals (MTB negative) was analyzed. Using the results obtained from this restrictive model, our main purpose was to understand better the dynamics of MTB transmission. Specifically, the occurrence of backward bifurcation was assessed in terms of the parameters γ, *p *and *q*, because this kind of bifurcation causes hysteresis-like behaviour. (Analytical results were obtained for *q *= 0 and *q *= 1.)

Backward bifurcation is encountered when the latent period is very large, that is, *γ < γ*^+^, a very low incubation rate. For instance, this kind of bifurcation occurs, considering the values given in Table 1, when γ<γ'=0.0128, where $\gamma '<\gamma_{+}=0.01588$ (*years*^-1^). Additionally, we must have a weak immune response, that is,*p > p_0_*, and quickly waning immunological memory,*q > q_c_*. Varying the re-infection (*q*) from 0 to 1 resulted in small variations with respect to *γ *in $\beta_{c}^{1}$, *i *= 0,1 (Figure 6), the lower bound of the transmission coefficient β at which backward bifurcation occurs. Small variations with respect to *γ *in $\beta_{c}^{q}$ are also found by varying super-infection (*p*) from 0.8 to 0.01; however, the order of magnitude of $\beta_{c}^{q}$ is increased (from 5 to 200, in *years*^-1^).

The long latency of MTB-positive persons plays a major role in MTB infection. From Figure 6, obtained using values given in Table 1, we conclude that:

(1) If latency is extremely long (for instance, *γ< 0.001 years*^-1^, which roughly corresponds to the probability of endogenous reactivation being less than 10% at age 100 *years*) then super-infection is needed to move latently infected persons more quickly into active TB, to maintain TB. Otherwise, virtually everyone would die naturally before they progressed and they would not transmit their TB. In this situation backward bifurcation promoting the hysteresis effect can maintain TB at an endemic level.

(2) If latency is not so long (for instance, *γ > 0.001 years*^-1^), backward bifurcation can occur. However, the range of transmission coefficients over which this kind of bifurcation can occur is small, and any external effects (for instance, immunosuppression due to diabetes or AIDS) that shortens the latent period can result in TB propagation at higher endemic levels than that predicted by backward bifurcation (see Figure 4). Additionally, an increase in the incubation rate decreases the threshold transmission coefficient (*β_0_*), which is an important aspect of MTB transmission. In developing countries, the decline in TB cases can be understood as *β < β_0_*, or near *β_0_*. The resurgence of TB cases after endemic transmission of HIV is due to *β *being larger than its threshold, which can be explained by a decrease in *β_0 _*that results in *R*_0 _> 1.

The model considered here does not produce a backward bifurcation under realistic conditions. However, understanding it as an approximation to a realistic model, we observed that this kind of bifurcation is relevant when the latency is very long. Backward bifurcation is indeed an important aspect that must be taken into account by health authorities when they act to interrupt MTB transmission, but this kind of bifurcation is strengthened when the incubation rate is very small. However, when this rate is not so small, backward bifurcation is not so prominent, and any factor (immunosuppression) that leads to an increase in the incubation rate results in a decrease in the threshold transmission coefficient *β_0_*, and potentially can result in *R_0 _> 1*. The disappearance of the break-point (the small positive solution assumes negative value) is another source of difficulty in controlling efforts because intervention must be so efficient in order to treat and isolate all infectious individuals. Moreover, the intervention must be continued, because the control of TB (trivial equilibrium) is unstable.

The results presented here approximate to the general model given by equation (1), which will be analyzed in a future paper. We will study the effects of long latency taking into account primary progression, super-infection and re-infection of MTB infection.

## Appendix A: Biology of TB

Tuberculosis (TB), a chronic infection usually affecting the lungs, claims more lives worldwide than any other infectious disease. It is caused by bacilli of the *Mycobacterium tuberculosis *(MTB) complex (*M. tuberculosis*, *M. bovis*, *M. africanum *and *M. microti*). MTB infects one third of the world's population and causes 8 million new cases of tuberculosis and approximately 2 million deaths each year. The two factors essential for its rapid spread are crowded living conditions and a population with little native resistance [26]. Despite a predominantly urban epidemiology, large tuberculosis outbreaks have also affected small communities [27].

In the vast majority of TB cases, this occurs through the forced expiration when a sputum smear-positive person coughs, sneezes, sings or speaks, aerosolizing respiratory droplets of varying size. Each cough for instance generates thousands (around 3000) of smaller particles in the order of 1 to 5 μ*m*, known as "droplet nuclei", which contain from one to three viable mycobacteria; talking for 5 minutes produces an equal number, and sneezing many more than that [26]. Transmission occurs when as few as one infectious particle is subsequently inhaled and deposited in the terminal alveoli of another person. The likelihood of this is a function of the concentration of droplet nuclei containing viable bacilli and the quantity of infected air that is inhaled. Thus, transmission is most likely to occur with prolonged contact in poorly ventilated environments [28].

In general, approximately 3-4% of infected individuals acquire active tuberculosis during the first year after tuberculin conversion, and a total of 5% to 15% do so thereafter. These estimates are based on heavy exposures during disease-prone periods of life. Persons infected with small inocula or during disease-resistant periods probably have much smaller risks, whereas the risk of progression in immunocompromised persons is greater. The likelihood of active disease developing varies with the intensity and duration of exposure. Persons with intense exposures are most at risk not only for infection but also for disease. It seems likely that active TB may ultimately develop in all persons with acquired immunodeficiency syndrome (AIDS) who are tuberculin positive [26].

Following transmission, infection occurs when MTB is phagocytosed by an alveolar macrophage. In the majority of cases the infectious process is arrested by the host's generation of a cell-mediated immune response. The sequence of events that ensues after phagocytosis of mycobacteria by macrophages involves the interaction of different T-cell subsets and their soluble products, as well as macrophages and other inflammatory cells. Mycobacteria have a variety of mechanisms that allow them to resist killing by inactivated macrophages, and thus to proliferate, essentially unchecked in this intracellular environment. At the same time, antigenic epitopes of the microorganism are processed for presentation to and recognition by T cells. This T-cell recognition and the subsequent release of cytokines leads to a state of macrophage activation and granuloma formation that, in the majority of cases, results in the suppression of mycobacterial proliferation. In over 95% of cases this immune response achieves the containment of MTB but does not completely eradicate it. This leaves the person infected with bacilli. During this latent phase, clinical manifestations of TB are mild and nonspecific and generally go undiagnosed [28].

Progression from latent to active disease is dictated by the balance between the virulent properties of the organism and the host defences. Infection remains controlled in 90% of infected persons, who will live their whole lives oblivious to the fact that they harbour viable mycobacteria. Overall, 5% of patients progress to disease within 2 years of infection, and another 5% do so during the reminder of their lives. These numbers are dramatically different in patients who have compromised cell-mediated immune systems. The likelihood of progression to active disease over the patient's lifetime is increased by a factor of 2 to 3 in persons with moderately immunocompromising conditions, e.g. diabetes. However, in patients with advanced human immunodeficiency virus (HIV) infection, progression to disease within 3 months of infection occurs in as many as one third of cases and the rate of subsequent progression is 7% to 10% per year [28].

Pulmonary TB may occur soon after infection (primary tuberculosis) or well after the primary focus have been contained (reactivation or postprimary disease). In the former case, failure of the host's immune response to contain the initial focus of infection results in progressive disease at the site of initial implantation. Reactivation is the result of proliferation of organisms in a previously dormant focus of infection, usually implanted during the primary dissemination phase of the infection, often in the distant past. In contrast to the primary case, which may occur anywhere in the lung, reactivation disease most often affects the apical posterior segments of the upper lobes, and is characterized by chronicity and progressive worsening [28].

Resistance to exogenous reinfection in the previously infected host is generally so great that new inocula are destroyed before significant multiplication occurs, with nearly all cases of active tuberculosis in such patients reflecting reactivation of latent foci. Although this is probably true in developed countries where the level of contagion is low, when contagion is high, exogenous reinfection is the rule [29]. Airflow in the apical posterior areas of the lung is low, but when inhaled droplet nuclei reach that location, as is more likely with high levels of contagion, bacillary multiplication will be favoured by the same local factors that enhance multiplication of blood-borne organisms [30,31]. Repeated inhalational exposures to tubercle bacilli maintain a high degree of tissue hypersensitivity and cellular immunity, making superinfection more difficult; however, when the airborne inoculum is large, or in immunocompromised hosts, super-infection can occur [26].

Tuberculosis is the prototype of infections that require a cellular immune response for their control. Although abundant antibodies are also produced during infection, these play no apparent role in host defence mechanisms. Two important populations of CD4 T cells can be identified in the response to mycobacterial infection: Th1 cells produce interleukin 2 (IL-2) and γ-interferon, which act as effector and regulatory elements in the cellular immune response; and Th2 cells produce IL-4, IL-5, IL-6 and IL-10, and provide help to B cells in the production of different immunoglobulins and the regulation of the humoral immune response [28]. The sustained immunity to new infection that follows natural infection is most likely due to the persistence of viable tubercle bacilli in the tissues with in vivo boosting. In tuberculin-positive persons, endogenous foci may reactivate repeatedly, and active CD4 lymphocyte surveillance is necessary to maintain quiescence [26]. Generally, the induction of T cell memory is characterized by a number of distinct phases [32]. Following antigen (Ag) priming, Ag-specific T cells undergo massive proliferation and clonal expansion followed by a concentration phase in which the vast majority of the activated cells are eliminated by apoptosis. During this primary response, memory T cells start to emerge and are maintained for extended periods either by retaining Ag, repeated stimulation/boosters, or homeostatic proliferation, hence providing a pool of cells that can rapidly respond to subsequent encounters with the pathogen [33].

There are several treatment regimens that have proven efficiencies in excess of 90%. All of them incorporate the basic principle of using multiple antimicrobial agents for prolonged periods of time administered under direct observation. The objectives of antituberculous chemotherapy are to decrease the infectivity of active cases rapidly, to reduce morbidity and mortality, and to effect a bacteriological cure [28].

## Appendix B: Analysis of the equilibrium points

We present an analysis of the model with respect to the equilibrium points taking into account super-infection (*p*) and a long period of incubation (*γ*^-1^).

### Disease free equilibrium

The equilibrium point $\left(\bar{s},\bar{e},\bar{y},\bar{z}\right)$ corresponding to the disease free (or trivial) steady state of the dynamical system (2) is given by *P*^0 ^= (1,0,0,0). To establish the stability of the equilibrium point *P*^0 ^we must evaluate the eigenvalues of the Jacobian matrix, related to system (2), taking into account the coordinates $\bar{s}=1$ and $\bar{e}=\bar{y}=\bar{z}=0$. Two eigenvalues are easily calculated, giving *λ_1 _= λ_2 _= -μ*, while the remaining *λ_3 _*and *λ_4 _*are obtained as the roots of the characteristic equation

(B.1) $$\Lambda^{0}\left(\lambda \right)=\lambda^{2}+c_{1}\lambda +c_{2}=0,$$

where, for *γ > 0*, the coefficients *c_1 _*and *c_2 _*are

$$\left\{ \begin{array}{l} c_{1}=2\mu +\gamma +\delta +\alpha \\ c_{2}=\left(\mu +\gamma \right)\left(\mu +\delta +\alpha \right)\left(1-R_{0}\right)\;, \end{array}\right.$$

and the basic reproduction ratio,

(B.2) $$R_{0}=\frac{\beta }{\beta_{0}}\;,$$

with β_0 _being the threshold transmission coefficient given by

(B.3) $$\beta_{0}=\frac{\left(\mu +\gamma \right)\left(\mu +\delta +\alpha \right)}{\gamma }\;.$$

According to the Routh-Hurwitz criteria the characteristic equation (B.1) has eigenvalues with negative real parts if and only if the coefficients *c_1 _*and *c_2 _*are positive. Since all parameters are positive, *c_2 _> 0 *always holds; while the sign of *c_2 _*depends on the value assumed by the parameter *γ. *For *γ > 0*, whenever *R_0 _< 1 *(or *β < β_0_*) then *c_2 _> 0*, and the trivial equilibrium point *P^0 ^*is locally asymptotically stable (LAS); otherwise, that is, whenever, *R_0 _> 1, P^0 ^*is unstable.

However, when *γ = 0*, the coefficients *c_1 _*and *c_2 _*simplify to

$$\left\{ \begin{array}{l} c_{1}=2\mu +\delta +\alpha \\ c_{2}=\mu \left(\mu +\delta +\alpha \right)\;, \end{array}\right.$$

and the trivial equilibrium point *P^0 ^*is always LAS since both inequalities *c_1 _> 0 *and *c_2 _> 0 *are satisfied.

### Disease at an endemic level

The non-trivial equilibrium point $\bar{P}=\left(\bar{s},\bar{e},\bar{y},\bar{z}\right)$ of the system (2), for *β≠0*, has coordinates given by

(B.4) $$\left\{ \begin{array}{l} \bar{s}=\frac{\mu +\alpha \bar{y}}{\mu +\beta \bar{y}}\\ \bar{e}=\frac{\mu +\delta +\alpha }{\gamma +p\beta \bar{y}}\bar{y}\\ \bar{z}=\frac{\delta }{\mu +q\beta \bar{y}}\bar{y}\;, \end{array}\right.$$

where the fraction of infectious individual at steady state ȳ is obtained as the positive roots of the equation $\beta \bar{y\times }Q^{*}\left(\beta \;\bar{y,\;}\right)=0$, where the third degree polynomial $Q^{*}\left(\beta \;\bar{y,\;}\right)$ is

(B.5) $$Q^{*}\left(\beta \;\bar{y,\;}\right)=a_{3}^{*}{\left(\beta \;\bar{y,\;}\right)}^{3}+a_{2}^{*}{\left(\beta \;\bar{y,\;}\right)}^{2}+a_{1}^{*}\left(\beta \;\bar{y,\;}\right)+a_{0}^{*}\;,$$

with the coefficients

(B.6) $$\left\{ \begin{array}{l} a_{3}^{*}=pq\\ a_{2}^{*}=p\left(\mu +\gamma \right)+q\left(\mu +\gamma +\delta +\alpha \right)+pq\left[\left(\mu +\alpha \right)-\beta \right]\\ a_{1}^{*}=\left(\mu +\gamma \right)\left(\mu +\delta +\alpha \right)\left(1-\frac{\alpha }{\beta_{0}}\right)+p\mu \left[\left(\mu +\gamma +\delta \right)-\beta \right]\\ \;\;\;\;\;\;\;\;\;\;\;+q\left(\mu +\gamma \right)\left(\mu +\delta +\alpha \right)\left(1-\frac{\beta }{\beta_{0}}-\frac{\delta }{\beta_{0}}\right)\\ a_{0}^{*}=\mu \left(\mu +\gamma \right)\left(\mu +\delta +\alpha \right)\left(1-\frac{\beta }{\beta_{0}}\right)\;, \end{array}\right.$$

and $\beta \;\bar{y}$ is the force of infection in the steady state. Note that one of the solution is $\;\bar{y}=0$, such that the trivial equilibrium point *P^0 ^*exists. When $\;\bar{y}\neq 0$, we can obtain the positive roots $\;\bar{y}$ of $Q^{*}\left(\beta \;\bar{y,\;}\right)$. The dimension of *x *is the same as *β*, that is, [*time*]^-1^, *β_0 _*is given by (B.3), and, according to (B.2), $\frac{\beta }{\beta_{0}}=R_{0}$.

Next we present analytical results with respect to the equilibrium points and their stability for two special cases: *q = 0 *and *q *= 1.

#### Determining equilibrium points

For *q *= 1, one of the roots of the polynomial (B.5) is $\;\bar{y}=-\frac{\mu }{\beta }$, a negative solution. Hence, the equation (B.5) can be reduced to the following second degree polynomial

(B.7) $$Q^{*}\left(\beta \;\bar{y,\;}\right)=a_{2}^{*}{\left(\beta \;\bar{y,\;}\right)}^{2}+a_{1}^{*}\left(\beta \;\bar{y,\;}\right)+a_{0}^{*}\;,$$

where the coefficients are given by

(B.8) $$\left\{ \begin{array}{l} a_{2}^{*}=p\\ a_{1}^{*}=p\left(\beta_{1}-\beta \right)\\ a_{0}^{*}=\gamma \left(\beta_{0}-\beta \right)\;. \end{array}\right.$$

For p > 0, *β_0 _*is given by equation (B.3) and the parameter *β_1 _*is defined as

(B.9) $$\beta_{1}=\frac{\left(\mu +\gamma +\delta +\alpha \right)}{p}+\left(\mu +\delta +\alpha \right)\;.$$

If *β_1 _*<*β_0_*, then $Q^{*}\left(\beta \;\bar{y,\;}\right)$ could have two positive roots. When *β_1 _*≥ *β_0_*, the polynomial $Q^{*}\left(\beta \;\bar{y,\;}\right)$ has zero or one positive solution: if *R_0 _< 1 *(or β < β_0_), we have only the trivial solution *P*^0^; otherwise, we have exactly one non-trivial equilibrium point $\bar{P}$. The case *p *= 0 is dealt with in the main text (case without super-infection).

In order to determine the number of positive solutions of the polynomial $Q^{*}\left(\beta \;\bar{y,\;}\right)$, given by equation (B.7), we assess the relative positions between *β_0 _*and $\beta_{1}$, by analyzing the function

$$f(p)\equiv \beta_{1}-\beta_{0}=\frac{\left(\mu +\gamma +\delta +\alpha \right)}{p}-\left(1-\frac{p}{p_{0}}\right)\;,$$

where, for *γ > 0*, we have the parameter *p*_0 _defined by

(B.10) $$p_{0}=\frac{\gamma \left(\mu +\gamma +\delta +\alpha \right)}{\mu \left(\mu +\delta +\alpha \right)}\;.$$

The case *γ = 0 *is dealt with in the main text (case without natural flow to TB).

Since *0 < p < 1*, when *p_0 _≥ 1*, we have *β_1 _≥ β_0_*. Hence, for *0 < p_0 _< 1 *and *p > p_0_*, we have *β_1 _< β_0_*.

The first condition *p_0 _< 1 *can be assessed by the function *g(γ)*, obtained as the difference between the numerator and the denominator of *p_0_*, given by

(B.11) $$g(\gamma )=\gamma^{2}+\left(\mu +\gamma +\alpha \right)\gamma -\mu \left(\mu +\delta +\alpha \right)<0\;.$$

The second degree polynomial g(γ) has two real roots, one positive and the other negative, assigned as *γ*_+ _and *γ*_-_, respectively. The positive root, that is, the critical incubation rate *γ*_+_, is given by

(B.12) $$\gamma_{+}=\frac{\left(\mu +\delta +\alpha \right)}{2}\left(\sqrt{1+\frac{4\mu }{\left(\mu +\delta +\alpha \right)}}-1\right)\;.$$

Therefore, for *γ *<*γ*_+ _we have g(γ) < 0 and *p_0 _< 1*; otherwise *p_0 _≥ 1*. At *γ *= *γ*_+ _we have *g(γ_+_) = 0 *and *p_0 _= 1*.

We observe that *γ*_+ _is a very small value [20] because *g(γ = μ) = μ^2 ^> 0 *implies μ > γ_+_. For instance, retaining only the first three terms of the expansion $\sqrt{1+\frac{4\mu }{\left(\mu +\delta +\alpha \right)}}$, we have

$$\gamma_{+}\approx \mu \left[1-\frac{\mu }{\left(\mu +\delta +\alpha \right)}\right]\;,$$

and *γ*_+ _→ *μ *for *δ *>> (*μ *+ *α*).

Let us now assess the second condition, *p > p_0_*. Firstly, when *p ≤ p_0 _*(weak force of secondary attack), we have *β_1 _≥ β_0_*, and the dynamics follows as the case *p_0 _≥ 1*, that is, if *R_0 _< 1 *(or *β < β_0_*), there exists only the trivial solution *P_0_*; otherwise, we have exactly one non-trivial equilibrium point.

However, the scenario is completely different for *p > p_0_*. In that case *β_0 _> β_1 _*and the number of positive solutions of the polynomial $Q^{*}\left(\beta \;\bar{y\;}\right)$, given by equation (B.7), depends on the value assigned to *β*. Thus, the polynomial (B.7) has one positive solution for *β > β_0 _*(*a_1 _< 0 *and *a_0 _< 0*) and zero or two positive solutions for *β_1 _< β < β_0 _*( *a_1 _< 0 *and *a_0 _> 0*). Moreover, for $\beta_{1}^{c}<\beta_{1}$, such that $\beta_{1}^{c}<\beta <\beta_{0}$, the equation (B.7) has exactly two positive roots. At $\beta =\beta_{1}^{c}$, we have $Q(\bar{\bar{x)}}=0$, where $\bar{\bar{x}}(\beta )$ is the minimum value of the polynomial $Q^{*}\left(\beta \;\bar{y\;}\right)$. Hence, the lower bound $\beta_{c}^{1}$ is given by

(B.13) $$\beta_{c}^{1}=\beta_{1}+\frac{2\gamma }{p}\left[\sqrt{1+\frac{p}{\gamma }\left(\beta_{0}-\beta_{1}\right)}-1\right]\;,$$

with $\beta_{1}<\beta_{c}^{1}<\beta_{0}$. Therefore, when $\beta <\beta_{c}^{1}$ we have $Q^{*}\left(\beta \;\bar{y\;}\right)>0$, so there are no positive solutions for the polynomial (B.7). At $\beta =\beta_{c}^{1}$ there exists a unique positive solution ${\bar{y}}_{1}^{*}$,

(B.14) $${\bar{y}}_{1}^{*}=\frac{\beta_{c}^{1}-\beta_{1}}{2\beta_{c}^{1}}=\frac{\frac{2\gamma }{p}\left[\sqrt{1+\frac{p}{\gamma }\left(\beta_{0}-\beta_{1}\right)}-1\right]}{2\beta_{c}^{1}},$$

while for $\beta_{1}^{c}<\beta <\beta_{0}$ there are two positive solutions for the equation (B.7), which will be denoted as ${\bar{y}}_{1}^{+}$ and ${\bar{y}}_{1}^{-}$, respectively, the large and the small roots. The large root ${\bar{y}}_{1}^{+}$ is monotonically increasing with β, while the small root ${\bar{y}}_{1}^{-}$ decreases monotonically assuming zero value at *β = β_0 _*and negative values for *β > β_0_*. The two equilibrium points are denoted by $P^{-}=\left({\bar{s}}_{1}^{-},{\bar{e}}_{1}^{-},{\bar{y}}_{1}^{-},{\bar{z}}_{1}^{-}\right)$ and $P^{+}=\left({\bar{s}}_{1}^{+},{\bar{e}}_{1}^{+},{\bar{y}}_{1}^{+},{\bar{z}}_{1}^{+}\right)$, where ${\bar{s}}_{1}^{•},\;\;{\bar{e}}_{1}^{•}$ and $\;{\bar{z}}_{1}^{•}$, given by equation (B.4), are calculated with $\;{\bar{y}}_{1}^{•}$, where • stands for + or -. We also define the turning value *R_p _*as

(B.15) $$R_{p}=\frac{\beta_{c}^{1}}{\beta_{0}}\;,$$

which is clearly less than 1, showing that an endemic situation can be found even when *R_0 _< 1*. The expressions for ${\bar{y}}_{1}^{+}$ and ${\bar{y}}_{1}^{-}$ and further results are presented in the main text.

In the absence of re-infection among recovered individuals (*q *= 0), the coefficients of the polynomial (B.7) are given by

$$\left\{ \begin{array}{l} a_{2}=p\left(\mu +\delta \right)\\ a_{1}=\left\{\left(\mu +\gamma \right)\left(\mu +\delta \right)+\mu \left[\alpha +p\left(\mu +\delta +\alpha \right)\right]\right\}\;\left(1-\frac{\beta }{\beta_{1}}\right)\\ a_{0}^{*}=\mu \left(\mu +\gamma \right)\left(\mu +\delta +\alpha \right)\left(1-\frac{\beta }{\beta_{0}}\right)\;, \end{array}\right.$$

where *R_0 _= β/β_0_*, with *β_0 _*given by the equation (B.3), and *β_1 _*given by

(B.16) $$\beta_{1}=\frac{\left(\mu +\gamma \right)\left(\mu +\delta \right)+\mu \left[\alpha +p\left(\mu +\delta +\alpha \right)\right]}{p\mu }\;,$$

resulting in

$$\beta_{0}-\beta_{1}=\frac{\mu^{2}\left(\mu +\delta +\alpha \right)}{p\mu \gamma }\left(p-p_{0}\right)\;.$$

In that case, the critical proportion *p_0 _*and the critical incubation rate *γ*_+ _are given by

(B.17) $$\left\{ \begin{array}{l} p_{0}=\frac{\gamma \left[\left(\mu +\gamma \right)\left(\mu +\delta \right)+\mu \alpha \right]}{\mu^{2}\left(\mu +\delta +\alpha \right)}\\ \gamma_{+}=\frac{\mu \left(\mu +\delta +\alpha \right)}{2\left(\mu +\delta \right)}\left(\sqrt{1+\frac{4\mu }{\left(\mu +\delta +\alpha \right)}}-1\right)\;, \end{array}\right.$$

while $\beta_{c}^{0}$ is now defined as

(B.18) $$\beta_{c}^{0}=\beta_{1}+\frac{2\gamma \left(\mu +\gamma \right)}{p\mu }\left[\sqrt{1+\frac{p\mu }{\gamma \left(\mu +\gamma \right)}\left(\beta_{0}-\beta_{1}\right)}-1\right]\;.$$

When *δ *>> (*μ + α*), we have

$$\gamma_{+}\approx \frac{\mu }{2}\left(\sqrt{5}-1\right)\approx 0.61803\times \mu ,$$

which is lower than the corresponding value for the case *q *= 1.

Summarizing, when *γ ≥ γ_+_*, there is exactly one positive solution for the polynomial $Q^{*}\left(\beta \;\bar{y\;}\right)$ given by equation (B.7). For *γ < γ*_+ _and *p > p_0_*, there are two positive solutions in the interval $\beta_{q}^{c}<\beta <\beta_{0}$, for *q *= 1,0. However, for *p ≤ p_0 _*a unique positive solution is obtained when γ < γ_+_.

#### Stability analysis

To proceed with the stability analysis of the non-trivial equilibrium point for the case *q *= 1, we use the compartment *w*, where *w = s + z*, according to the dynamical system (3) in terms of the variables (*w*,*e*,*y*). This dynamical system has the non-trivial equilibrium point $\bar{P}=\left(\bar{w},\bar{e},\bar{y}\right)$ where the coordinates are

$$\bar{w}=\bar{s}+\bar{z}=\frac{\mu +\left(\delta +\alpha \right)\bar{y}}{\mu +\beta \bar{y}},$$

$\bar{\text{e}}$ is given by equation (B.4) and $\bar{y}$ is the solution of the polynomial $Q^{*}\left(\beta \;\bar{y\;}\right)$, given by equation (B.7).

The Jacobian matrix **J **related to the system (3), after some re-arrangements, is

$$\text{J}=\left[\begin{array}{ccc} -\left(\mu +\beta y\right) & 0 & -\mu \frac{1-w}{y}\\ \beta y & -\left[\left(\mu +\gamma \right)+\gamma +p\beta y\right] & \left(\mu +\gamma \right)\frac{e}{y}\\ 0 & \gamma +p\beta y & -\gamma \frac{e}{y} \end{array}\right]\;.$$

The characteristic polynomial, corresponding to the Jacobian **J **evaluated at the non-trivial equilibrium point $\bar{P}=\left(\bar{w},\bar{e},\bar{y}\right)$, is given by

$$\Lambda \left(\lambda \right)=\lambda^{3}+b_{2}\lambda^{2}+b_{1}\lambda +b_{0}=0,$$

where the coefficients are

(B.19) $$\left\{ \begin{array}{l} b_{2}=2\mu +\left(p+1\right)\beta \bar{y}+\gamma \left(1+\frac{\bar{e}}{\bar{y}}\right)\\ b_{1}=\mu \left[\left(\mu +\beta \bar{y}\right)+\left(\gamma +p\beta \bar{y}\right)+\gamma \frac{\bar{e}}{\bar{y}}\right]+\frac{b_{0}}{\mu }\\ b_{0}=\mu \beta \left[{\left(\gamma +p\beta \bar{y}\right)}^{2}+\left(\gamma -p\mu \right)\left(\mu +\delta +\alpha \right)\right]\frac{\bar{y}}{\left(\gamma +p\beta \bar{y}\right)}\;\;\;. \end{array}\right.$$

According to the Routh-Hurwitz criteria, this third degree polynomial has all roots with negative real parts if *b_2 _*> 0 (always true for a positive equilibrium point), *b*_0 _> 0 and *b*_2_*b*_1 _- *b*_0 _> 0. The last condition, after some calculations, can be written as

$$\begin{array}{l} b_{2}b_{1}-b_{10}=\mu \left[2\mu +\left(p+1\right)\beta \bar{y}+\gamma \left(1+\frac{\bar{e}}{\bar{y}}\right)\right]\left[\left(\mu +\beta \bar{y}\right)+\left(\gamma +p\beta \bar{y}\right)+\gamma \frac{\bar{e}}{\bar{y}}\right]\\ \;\;\;\;\;\;\;\;\;\;\;\;\;\;\;\;\;\;\;\;\;\;\;\;\;\;\;\;\;+\left[\mu +\left(p+1\right)\beta \bar{y}+\gamma \left(1+\frac{\bar{e}}{\bar{y}}\right)\right]\frac{b_{0}}{\mu }\;, \end{array}$$

showing that whenever *b*_0 _> 0, the condition *b*_2_*b*_1 _- *b*_0 _> 0 is automatically satisfied (that is also true for *b*_1 _> 0). This confirms the conjecture provided in [34]. Therefore, local stability is determined by the sign of *b*_0_. Let us study the sign of the coefficient *b*_0 _in equation (B.19). The sign of *b*_0 _is determined by the term between square brackets, which can be written as a polynomial equation

$$F\left(\bar{y}\right)={\left(\gamma +p\beta \bar{y}\right)}^{2}-\left(p\mu -\gamma \right)\left(\mu +\delta +\alpha \right)=0.$$

Let us analyze the case *γ < pμ *First, let us determine a positive root of $F\left(\bar{y}\right),\bar{y}=\theta$, such that yields *b*_0 _= 0, by solving

$$F\left(\theta \right)={\left(\gamma +p\beta \theta \right)}^{2}-\left(p\mu -\gamma \right)\left(\mu +\delta +\alpha \right)=0.$$

When *0 < γ <ζ(p)*, the polynomial $F\left(\bar{y}\right)$, has one positive real root *θ*_+_, given by

$$\theta_{+}=\frac{1}{p\beta }\sqrt{\left(p\mu -\gamma \right)\left(\mu +\delta +\alpha \right)}-\gamma ,$$

where ζ(*p*), the positive solution of *G(γ) = γ^2 ^*+ (*μ + γ + α*)*γ - pμ*(*μ + δ + α*) = 0, is an increasing function defined by

$$\zeta \left(p\right)=\frac{\mu \left(\mu +\delta +\alpha \right)}{2}\left(\sqrt{1+\frac{4\mu }{\left(\mu +\delta +\alpha \right)}}-1\right)\;,$$

with *ζ(0) = 0 *and *ζ(1) = γ_+_*, given by equation (B.12). In this interval, $F\left(\bar{y}\right)<0$ for $\bar{y}<\theta_{+}$, and $F\left(\bar{y}\right)>0$ for $\bar{y}>\theta$. When *γ > ζ(p)*, we have no positive real root, and $F\left(\bar{y}\right)>0$. Clearly, $F\left(\bar{y}\right)>0$ for *γ ≥ pμ*. Summarizing, we have

$$F\left(\bar{y}\right)\;\;\;\;\left\{ \begin{array}{l} \;\;>0\text{​}\text{​}\text{​}\text{​}\text{​}\text{​};\;\;\;\;\;\;\;\;\;\;\;\;\;\gamma \geq p\mu \\ \;\;>0;\;\;\;\;\;\;\;\;\;\;\;\;\;\zeta \left(p\right)<\gamma <p\mu \\ \;\;>0;\;\;\;\;\;\;\;\;\;\;\;\;\;\;\left\{ \begin{array}{l} 0<\gamma <\zeta \left(p\right)\\ \bar{y}>\theta_{+} \end{array}\right.\\ \;\;<0;\;\;\;\;\;\;\;\;\;\;\;\;\;\;\;\left\{ \begin{array}{l} 0<\gamma <\zeta \left(p\right)\\ \bar{y}<\theta_{+}\;\;. \end{array}\right. \end{array}\right.$$

We have *b_0 _> 0 *whenever $F\left(\bar{y}\right)>0$. Since *0 ≤ p ≤ 1*, we have *ζ(p) ≤ γ_+_*. It is easy to show that *ζ(p) ≤ pμ*. On the other hand, from *μ > γ*_+_, for sufficiently higher *p *we have *p μ ≥ γ*_+_.

As we have shown in foregoing section, when γ < γ_+ _and *p > p_0_*, we have two positive solutions in the interval $\beta_{c}^{1}<\beta <\beta_{0}$, which were named ${\bar{y}}_{1}^{-}$ and ${\bar{y}}_{1}^{+}$, where ${\bar{y}}_{1}^{-}<{\bar{y}}_{1}^{+}$. By the fact that $F\left(\bar{y}\right)$ changes from negative to positive values at $\bar{y}=\theta_{+}$, we must position θ_+ _with respect to ${\bar{y}}_{1}^{-}$ and ${\bar{y}}_{1}^{+}$. In order to do this, we evaluate the second degree polynomial $Q^{*}\left(\beta \;\bar{y\;}\right)$, with positive coefficient for ${\left(\beta \;\bar{y\;}\right)}^{2}$ and given by equation (B.7), at $\beta \;\bar{y\;}=\beta \;\theta_{+}$, and determine $Q^{*}\left(\beta \;\theta_{+}\right)$, which is

$$Q\left(\beta \theta_{+}\right)=-\frac{1}{p}\sqrt{\left(p\mu -\gamma \right)\left(\mu +\delta +\alpha \right)}\left[p\left(\beta -\beta_{1}\right)+2\gamma -2\sqrt{\left(p\mu -\gamma \right)\left(\mu +\delta +\alpha \right)}\right]\;,$$

where *β_1 _*is given by equation (B.9). First, we must have *γ < pμ*, which includes the range *γ < γ_+ _*for sufficiently higher *p*. Second, the condition *p_0 _< p < 1 *establishes that *β_1 _< β_0_*, with *β_0 _*being given by equation (B.3). Finally, with $\beta_{c}^{1}$ being given by equation (B.13) and $\beta_{1}<\beta_{c}^{1}$, we have two positive solutions in the interval $\beta_{1}^{c}<\beta <\beta_{0}$. When we have two positive solutions, we will show that $Q^{*}\left(\beta \;\theta_{+}\right)<0$ and ${\bar{y}}_{1}^{-}<\theta_{+}<{\bar{y}}_{1}^{+}$, whenever the term between square brackets is positive. Initially, we rewrite equation (B.13) as

$$p\left(\beta_{c}^{1}-\beta_{1}\right)+2\gamma =2\sqrt{\gamma^{2}+p\gamma \left(\beta_{0}-\beta_{1}\right)},$$

and substituting *β_0 _*and *β_1_*, given by equations (B.1) and (B.9), respectively, we have

$$p\left(\beta_{c}^{1}-\beta_{1}\right)+2\gamma =2\sqrt{\left(p\mu -\gamma \right)\left(\mu \delta ++\alpha \right)}.$$

Now, in the term between square brackets of $Q^{*}\left(\beta \;\theta_{+}\right)$, we substitute *β *by its lower bound $\beta_{1}^{c}$, in order to have

$$\begin{array}{l} p\left(\beta -\beta_{1}\right)+2\gamma -2\sqrt{\left(p\mu -\gamma \right)\left(\mu \delta ++\alpha \right)}\geq \\ \geq p\left(\beta_{c}^{1}-\beta_{1}\right)+2\gamma -2\sqrt{\left(p\mu -\gamma \right)\left(\mu \delta ++\alpha \right)}=0\;, \end{array}$$

according to the last result. Therefore, for $\beta_{1}^{c}<\beta <\beta_{0}$, we have $Q^{*}\left(\beta \;\theta_{+}\right)<0$. Hence, $F\left({\bar{y}}_{1}^{-}\right)<0$ and $F\left({\bar{y}}_{1}^{+}\right)<0$, which permit us to conclude that the small equilibrium ${\bar{y}}_{1}^{-}$ is unstable (b_0 _< 0), and the large equilibrium ${\bar{y}}_{1}^{+}$ is LAS (b_0 _> 0).

In the case of a unique non-trivial equilibrium point (*γ ≥ γ*_+_, or *γ < γ*_+ _and *p ≤ p_0_*), we have b_0 _> 0. Hence the stability of the positive equilibrium point is given by the basic reproduction number *R*_0 _given by equation (B.2): when *R*_0 _> 1 (or *β *>*β*_0_), the non-trivial equilibrium point $\bar{P}=\left(\bar{s},\bar{e,\;}\bar{y},\bar{z}\right)$ is LAS. When *R*_0 _≤ 1 (or *β *≤ *β*_0_), we have $\bar{y}\leq 0$, hence *b_0 _≤ 0*.

We established that the so-called backward bifurcation occurs only over a very narrow range *γ < γ*_+_, remembering that *γ_+ _< μ*. Otherwise we have the well-known forward bifurcation based on the threshold value *β_0_*. The restriction on the incubation rate γ establishes that the 'strange (or catastrophic) bifurcation' [35] occurs only when the onset of the disease is less frequent than the death of humans, or, in other words, the period of time elapsed to progression to the disease is greater than the survival time of humans.

## Competing interests

The authors declare that they have no competing interests.

## Authors' contributions

Both authors conceived, analyzed and discussed the model. All authors read and approved the final manuscript.
